# Gcn5-Related *N-*Acetyltransferases (GNATs) With a Catalytic Serine Residue Can Play Ping-Pong Too

**DOI:** 10.3389/fmolb.2021.646046

**Published:** 2021-04-12

**Authors:** Jackson T. Baumgartner, Thahani S. Habeeb Mohammad, Mateusz P. Czub, Karolina A. Majorek, Xhulio Arolli, Cillian Variot, Madison Anonick, Wladek Minor, Miguel A. Ballicora, Daniel P. Becker, Misty L. Kuhn

**Affiliations:** ^1^Department of Chemistry and Biochemistry, San Francisco State University, San Francisco, CA, United States; ^2^Department of Chemistry and Biochemistry, Loyola University Chicago, Chicago, IL, United States; ^3^Department of Molecular Physiology and Biological Physics, University of Virginia, Charlottesville, VA, United States; ^4^Center for Structural Genomics of Infectious Diseases (CSGID), University of Virginia, Charlottesville, VA, United States

**Keywords:** Gcn5-related *N-*acetyltransferase, ping-pong kinetic mechanism, acetylation, acetyltransferase, docking, enzyme mechanism, *Pseudomonas aeruginosa*

## Abstract

Enzymes in the Gcn5-related *N-*acetyltransferase (GNAT) superfamily are widespread and critically involved in multiple cellular processes ranging from antibiotic resistance to histone modification. While acetyl transfer is the most widely catalyzed reaction, recent studies have revealed that these enzymes are also capable of performing succinylation, condensation, decarboxylation, and methylcarbamoylation reactions. The canonical chemical mechanism attributed to GNATs is a general acid/base mechanism; however, mounting evidence has cast doubt on the applicability of this mechanism to all GNATs. This study shows that the *Pseudomonas aeruginosa* PA3944 enzyme uses a nucleophilic serine residue and a hybrid ping-pong mechanism for catalysis instead of a general acid/base mechanism. To simplify this enzyme’s kinetic characterization, we synthesized a polymyxin B substrate analog and performed molecular docking experiments. We performed site-directed mutagenesis of key active site residues (S148 and E102) and determined the structure of the E102A mutant. We found that the serine residue is essential for catalysis toward the synthetic substrate analog and polymyxin B, but the glutamate residue is more likely important for substrate recognition or stabilization. Our results challenge the current paradigm of GNAT mechanisms and show that this common enzyme scaffold utilizes different active site residues to accomplish a diversity of catalytic reactions.

## Introduction

Acetyltransferases are fascinating enzymes found across all domains of life. They are critically important for various cellular functions including those of anabolic and catabolic pathways, cell wall modification, xenobiotic metabolism, and antibiotic drug resistance (Sim et al., 2014; Hentchel and Escalante-Semerena, 2015; Zhang et al., 2017; Burckhardt and Escalante-Semerena, 2020; Sharma et al., 2020). Their seemingly simple ability to catalyze the transfer of an acetyl moiety from a donor molecule to an acceptor molecule is compounded by the diversity of structural scaffolds, active site residues, and kinetic mechanisms that they utilize. In general, acetyltransferases are grouped into a variety of families based on their structural folds and the types of substrates they acetylate. Some well-studied structural scaffolds of bacterial acetyltransferases include the hexapeptide repeat fold, arylamine *N*-acetyltransferase (NAT) fold, and the Gcn5-related *N-*acetyltransferase (GNAT) fold. All three of these families of acetyltransferases play intricate roles in bacterial cellular processes, and therefore are worthy of dedicated study.

We have been working to improve structural and functional coverage of uncharacterized bacterial GNATs so that we may (1) learn more about the diversity of structures and functions of these enzymes and (2) improve the annotation of sequenced genomes. GNATs have a characteristic V-like splay at the core of their structures. They contain both a donor site where acetyl-coenzyme A (AcCoA) or another acyl donor binds and an acceptor site. While the donor site is relatively conserved, the residues that comprise the acceptor site vary widely and contribute to substrate specificity. The majority of GNATs that have been functionally characterized perform *N-*acetylation of primary amines (Vetting et al., 2005b; Favrot et al., 2016; Burckhardt and Escalante-Semerena, 2020), but a few examples of *O-*acetylation of hydroxyl groups exist (Daigle et al., 1999; Hegde et al., 2001). Recent studies have also shown that the GNAT fold has been repurposed by some organisms to catalyze decarboxylation instead of acyl transfer, thus highlighting the sheer diversity of reaction capabilities of members of this superfamily of proteins (Skiba et al., 2020).

The primary chemical mechanism described for GNATs is a general acid/base mechanism that proceeds through the use of a tyrosine residue as a general acid and a glutamate residue as a general base. The base abstracts a proton from the conjugate acid of the acceptor amine, which enables the acceptor substrate to perform a nucleophilic attack on the acetyl donor; the general acid then protonates the thiolate anion of CoA. While this is the generally accepted mechanism, there have been examples of GNATs where a catalytic base could not be identified. In those cases, a water molecule or proton wire was proposed to deprotonate the acceptor substrate (Hickman et al., 1999). It has even been suggested that the approach of the acceptor substrate into the active site lowers the pKa of the acceptor amine or enables it to become deprotonated without use of a general base (Montemayor and Hoffman, 2008; Oda et al., 2010). Typical residues that can act as general acids are tyrosine and cysteine, while residues that can act as general bases include histidine, glutamate, and aspartate. While some reports have suggested serine can act as a general acid, its pKa is too high to serve in this capacity. Instead, it would more likely act as a nucleophile if it participates in the chemical mechanism. Therefore, its presence at the typical location of the general acid in a GNAT active site suggests the utilization of an alternative chemical mechanism. Furthermore, even when a tyrosine residue is placed appropriately to act as a general acid in a GNAT active site, it is not a definitive indication that it acts as an acid or that the enzyme utilizes a general acid/base mechanism (Draker and Wright, 2004).

Two main types of kinetic mechanisms for GNATs have been proposed: a direct transfer or sequential mechanism, and a ping-pong mechanism. In a direct transfer mechanism the acetyl group is transferred directly from AcCoA to the acceptor substrate, whereas in a ping-pong mechanism the acetyl group is transferred first to the enzyme to form an acyl-enzyme intermediate and then from the enzyme to the acceptor substrate. For a ping-pong mechanism to occur, a nucleophilic residue such as a cysteine or serine must be present at an appropriate position in the active site. Several GNATs have been described that have cysteine residues in their active sites (Vetting et al., 2005a; Charlop-Powers et al., 2012; Majorek et al., 2013). However, all studies that have examined the criticality of these cysteines have shown they are not likely to be directly involved in catalysis. A few studies have reported a serine residue in GNAT active sites, but the role of this residue and potential involvement in the kinetic mechanism of the enzyme has only been suggested (Vetting et al., 2005a). While a ping-pong mechanism has been widely proposed as a probable kinetic mechanism for GNATs in the literature, to our knowledge only one example of a GNAT with evidence to support this mechanism has been described (Sakamoto et al., 1998).

Previously, we determined the structure of the PA3944 GNAT enzyme from *Pseudomonas aeruginosa* and found that it could acetylate polymyxin antibiotics, specifically the 3-Dab residue on polymyxin B and on colistin (Czub et al., 2018). This former study laid the foundation for us to further explore key residues that may be important for PA3944 activity, but the complexities of the polymyxin B substrate complicated our interpretation of kinetic data as well as our ability to obtain a liganded crystal structure. Therefore, in this study we report the synthesis, kinetic characterization, and docking of a simpler designed substrate analog of polymyxins, *N-*(2-aminoethyl)-*N-*methyloctanamide (NANMO). We also investigated the importance of two key residues in the active site (S148 and E102) and propose a chemical and kinetic mechanism for this enzyme that is contrary to nearly all characterized GNATs reported in the literature. Our results highlight key characteristics of certain GNATs that will be useful for identifying homologs that may exhibit similar mechanistic behaviors. Moreover, these results help to define a new sub-group of GNATs, which will improve their functional characterization and genome annotation.

## Materials and Methods

### Materials

Polymyxin B, coenzyme A trilithium salt, and acetyl coenzyme A trilithium salt were purchased from Millipore Sigma. All other reagents were purchased at the highest quality available.

### Experimental

All solvents were distilled prior to use and all reagents were used without further purification unless otherwise noted. All synthetic reactions were conducted under an atmosphere of nitrogen. Silica gel 60 Å, 40-75 μm (200 × 400 mesh), was used for column chromatography. Aluminum-backed silica gel 200 μm plates were used for TLC. ^1^H NMR spectra were obtained using a 500 MHz spectrometer with tetramethylsilane (TMS) as the internal standard. ^13^C NMR spectra were obtained using a 75 or 125 MHz spectrometer. NMR spectra were processed using the Mnova NMR software program produced by Mestrelab Research. The purity of all compounds was determined to be ≥95% unless otherwise noted by high performance liquid chromatography (HPLC) employing a mobile phase *A* = 5% acetonitrile in water and a mobile phase *B* = 0.1% TFA in acetonitrile with a gradient of 60% B increasing to 95% over 10 min, holding at 95% B for 5 min, then returning to 60% B and holding for 5 min. HRMS spectra were measured on a TOF instrument by electrospray ionization (ESI).

### *N*-(2-Aminoethyl)-*N*-Methyloctanamide Hydrochloride (NANMO) Synthesis and Purification

**FIGURE 1:**
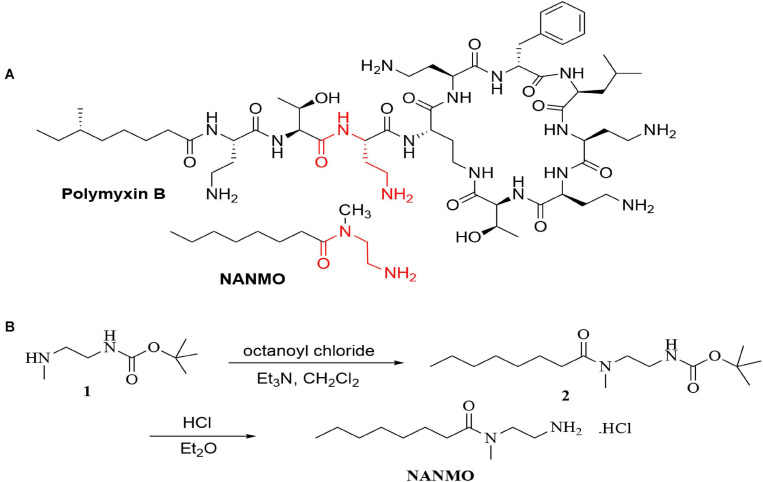
**(A)** The structures of polymyxin B and NANMO. The 3-Dab of polymyxin B is shown in red. The common substructures of NANMO and polymyxin B are shown. **(B)** The synthesis of NANMO.

Triethylamine (0.40 mL, 2.86 mmol) was added to a stirred solution of *N*-(tert-butoxycarbonyl)-*N*-methylethylenediamine (250 mg, 1.43 mmol) in methylene chloride (7.5 mL) at 0°C in an ice bath followed by the dropwise addition of octanoyl chloride (0.3 mL, 1.72 mmol). The reaction was stirred at room temperature for 24 h. The resultant solution was washed successively with water (3 mL), 1 M aqueous HCl (3 mL) and 1 M aqueous NaOH (3 mL). The dichloromethane layer was dried over sodium sulfate and the solvent was evaporated under reduced pressure to give *tert*-butyl (2-(*N*-methyloctanamido)ethyl)carbamate (338 mg, 79% yield) as a colorless oil. Without further purification, *tert*-butyl (2-(*N*-methyloctanamido)ethyl)carbamate (338 mg, 1.13 mmol) was dissolved in diethyl ether (0.6 mL). Then, 2.0 M HCl in diethyl ether (0.2 mL, 4.11 mmol) was added and the reaction mixture was stirred 2 h at room temperature. The resulting white precipitate was filtered off, washed with diethyl ether and dried under dry nitrogen to give *N*-(2-aminoethyl)-*N*-methyloctanamide (NANMO) as the hydrochloride salt (102 mg, 45 % yield) (Figure 1B). ^1^H NMR (500 MHz, D_2_O, doubling of some peaks due to amide rotamers) δ 3.67 (t, *J* = 6.9 Hz, 0.2H), 3.59 (t, *J* = 6.1 Hz, 1.8H), 3.18 (t, *J* = 6.8 Hz, 0.3H), 3.12 (t, *J* = 6.1 Hz, 1.7H), 3.03 (s, 2.5H), 2.86 (s, 0.5H), 2.39–2.33 (m, 2H), 1.50 (h, *J* = 7.3, 6.9 Hz, 2H), 1.29–1.15 (m, 8H), 0.79 (t, 3H). ^13^C NMR (126 MHz, MeOD, doubling of some peaks due to amide rotamers) δ 176.28, 176.02, 49.15, 48.13, 47.96, 47.79, 47.62, 47.45, 47.28, 47.11, 45.46, 37.77, 35.57, 35.55, 35.15, 32.97, 32.41, 31.53, 31.47, 29.02, 28.94, 28.88, 28.74, 27.38, 25.25, 24.56, 22.29, 22.28, 13.03, 13.01. HRMS (ESI) calcd (MH^+^) C_1__1_H_2__4_N_2_O: 201.1967, Obs: 201.1960 (100.00) (Scans of NANMO NMR spectra in Supplementary Material: ^1^H NMR, Supplementary Figures S1, S2; ^13^C NMR, Supplementary Figures S3, S4).

### Clones and Site Directed Mutagenesis

The clone containing the wild-type *pa3944* gene as previously described (Czub et al., 2018) was used as the template for constructing E102A and S148A point mutations. These mutants were created using the QuikChange site-directed mutagenesis kit (Stratagene) and the procedure previously described (Majorek et al., 2017). All correct sequences of point mutants were confirmed by DNA sequencing (Genewiz).

### Protein Expression and Purification

The wild-type and mutant PA3944 proteins were heterologously expressed and purified using the same procedures previously described (Czub et al., 2018). All proteins for kinetic analysis retained the N-terminal polyhistidine tag, but the tag was removed for crystallization trials. SDS-PAGE was used to confirm that all proteins were purified to near homogeneity.

### Steady-State Enzyme Kinetics Assays and Kinetic Mechanism Model Fitting

All enzyme assays and substrate saturation curves for the wild-type and mutant enzymes were performed exactly as described (Czub et al., 2018) with varying concentrations of NANMO (0–3.5 mM for WT, 0–2.5 mM for E102A) and polymyxin B (0–6 mM for WT, 0–10 mM for E102A). A solution of the hydrochloride salt of NANMO was prepared as a 100 mM stock solution in water. To determine the model that best described the kinetic mechanism of the PA3944 enzyme, a series of substrate saturation curves for NANMO (0–3.5 mM) and polymyxin B (0–6 mM) with the WT enzyme were produced at different concentrations of AcCoA (0.1, 0.25, 0.5 and 1 mM). At least two biological replicates were collected for all enzyme kinetics assays. Models that were tested and fitted to the kinetic data included the following, and the goodness of fit of each model was assessed using Akaike’s Information Criterion (AICc) values as described previously (Filippova et al., 2015).

Random bisubstrate steady-state equation:

$$V=\frac{V_{m}\,\left[A\right]\,\left[B\right]}{K_{a}\,K_{b}+K_{a}\,\left[B\right]+K_{b}\,\left[A\right]+\left[A\right]\,\left[B\right]}$$

$$\text{Where }V_{m}={\left[E\right]}_{t}\,k_{p}$$

Ordered AB bisubstrate steady-state equation:

$$V=\frac{V_{m}\,\left[A\right]\,\left[B\right]}{K_{a}\,K_{b}+K_{b}\,\left[A\right]+\left[A\right]\,\left[B\right]}$$

$$\text{Where }V_{m}={\left[E\right]}_{t}\,k_{p}$$

Ordered BA bisubstrate steady-state equation:

$$V=\frac{V_{m}\,\left[A\right]\,\left[B\right]}{K_{a}\,K_{b}+K_{a}\,\left[B\right]+\left[A\right]\,\left[B\right]}$$

$$\text{Where }V_{m}={\left[E\right]}_{t}\,k_{p}$$

Ping-pong bisubstrate steady-state equation:

$$V=\frac{V_{m}\,\left[A\right]\,\left[B\right]}{K_{a}\,\left[B\right]+K_{b}\,\left[A\right]+\left[A\right]\,\left[B\right]}$$

$$\text{Where }V_{m}={\left[E\right]}_{t}\,k_{\mathrm{cat}}=\frac{k_{p}\,k_{q}}{k_{q}+k_{p}}$$

Hybrid ping-pong bisubstrate steady-state equation:

$$V=\frac{n_{1}\,\left[A\right]\,{\left[B\right]}^{2}+n_{2}\,{\left[A\right]}^{2}\,\left[B\right]+n_{3}\,\left[A\right]\,\left[B\right]}{d_{1}\,\left[A\right]\,{\left[B\right]}^{2}+d_{2}\,{\left[A\right]}^{2}\,\left[B\right]+d_{3}\,{\left[B\right]}^{2}+d_{4}\,\left[A\right]\,\left[B\right]+d_{5}\,{\left[A\right]}^{2}+d_{6}\,\left[B\right]+d_{7}\,\left[A\right]}$$

Where $n_{1}=\frac{k_{7}}{\varepsilon_{a}}$; *n_2=1*; $n_{3}=\frac{k_{8}}{\varepsilon_{a^{\prime }}}$; $d_{1}=\frac{k_{7}}{\varepsilon_{a}\,V_{m\,S\,e\,q}}$; $d_{2}=\frac{1}{V_{m\,P\,P}};d_{3}=\frac{k_{7}}{\varepsilon_{a}\,\varepsilon_{a^{\prime }}}$; $d_{4}=\left(\frac{1}{\varepsilon_{a}}\right)\,\left(1+G\,\frac{k_{7}}{\varepsilon_{b}}\right)+\left(\frac{k_{8}}{\varepsilon_{a^{\prime }}\,V_{m\,P\,P}}\right)$; $d_{5}=\frac{1}{\varepsilon_{b}}$; $d_{6}=\frac{k_{8}}{\varepsilon_{a}\,\varepsilon_{a^{\prime }}}$; $d_{7}=\frac{k_{8}}{\varepsilon_{b}\,\varepsilon_{a^{\prime }}}$; $\frac{1}{V_{m\,P\,P}}=\frac{k_{6}+k_{3}}{k_{3}\,k_{6}}$; $\frac{1}{V_{m\,S\,e\,q}}=\frac{k_{11}+k_{6}}{k_{6}\,k_{11}}$; $\frac{1}{\varepsilon_{a}}=\frac{k_{2}+k_{3}}{k_{1}\,k_{3}}$; $\frac{1}{\varepsilon_{b}}=\frac{k_{6}+k_{5}}{k_{4}\,k_{6}}$; $\frac{1}{\varepsilon_{a^{\prime }}}=\frac{k_{10}+k_{11}}{k_{9}\,k_{11}}$; $G=\frac{k_{5}}{k_{6}+k_{5}}$; $K_{d_{B}}=\frac{k_{8}}{k_{7}}$.

V_*m*__*Seq*_ is the maximal velocity of the sequential path, V_*mPP*_ is the maximal velocity of the ping-pong path, and ε_*a*_, ε_*b*_, and ε_*a*__’_ represent an analog of a catalytic efficiency at the formation and destruction of species EA, EB, and EAB, respectively. See Supplementary Scheme S1 for further details and the derivation of the equation.

### Protein Crystallization, Data Collection, and Structure Determination

The PA3944 WT and E102A mutant proteins were crystallized using the sitting drop vapor diffusion technique. Crystallization plates (3-Well Midi, Swissci) were set using a Mosquito crystallization robot (TTP Labtech) and incubated at 16°C. Prior to crystallization, powdered CoA was added to the PA3944 E102A protein to a final concentration of 5 mM, while AcCoA and (R)-3-(2-chloroacetamido)-4-[((S)-1-methoxy-1-oxo-3-phenylpropan-2-yl)amino]-4-oxobutanoic acid were added to the PA3944 WT protein (5 mM each). Aliquots of 0.2 μL of protein at a concentration of 10 mg/mL in buffer (100 mM Tris-HCl pH 7.5 and 150 mM NaCl) were mixed with 0.2 μL aliquots of reservoir solution (MCSG1 screen, well C11: 100 mM Tris-HCl pH 7.0, 200 mM calcium acetate monohydrate, 20% w/v PEG 3000). Crystals were harvested and mounted over 1 M sodium chloride solution for 15–20 min (a slow dehydration technique) and then flash-cooled without any additional cryoprotection.

Diffraction data were collected at the SBC-CAT 19-BM and LS-CAT 21-ID-G beamlines at the Advanced Photon Source (Argonne National Laboratory). Data collection was performed at 100 K, using a 0.979 Å wavelength. Collected data were processed, integrated and scaled using HKL-3000 (Otwinowski and Minor, 1997; Minor et al., 2006). The structures were determined by molecular replacement using a previously determined structure of the WT PA3944 protein (PDB ID: 6EDD) as the template. Structure determination and refinement were performed using HKL-3000 coupled with MOLREP (Vagin and Teplyakov, 2010), REFMAC (Murshudov et al., 2011), Coot (Emsley et al., 2010), and other programs from the CCP4 package (Winn et al., 2011). The refinement process followed the most recent guidelines (Shabalin et al., 2018). The protein models were placed in the standard position in the unit cell using the ACHESYM server (Kowiel et al., 2014). TLS groups were determined by the TLS Motion Determination Server (Painter and Merritt, 2006) during the refinement process. The LabDB database (Cooper et al., 2021) was used to track all experimental steps (purification, crystallization, data collection and structure determination/refinement). Molstack, an internet platform (Porebski et al., 2018), was used for interactive visualization of the PA3944 models and their respective fit to electron density maps. Diffraction images have been deposited into the Integrated Resource for Reproducibility in Macromolecular Crystallography (Grabowski et al., 2016) with the following identifiers: doi: 10.18430/m37kps and doi: 10.18430/m37kpp for 7KPS and 7KPP, respectively. The crystal structures have been deposited into the Protein Data Bank (PDB) with the following identification codes: PDB IDs: 7KPP and 7KPS for the PA3944 E102A mutant and PA3944 WT enzyme, respectively. The structure quality (fit to the electron density map) for both structures can be inspected interactively at https://molstack.bioreproducibility.org/project/view/w7qpz5GeCt6pr9ikpoDi/.

### Molecular Docking

A molecular model of NANMO was developed using the Molecular Operating Environment (MOE) computational suite’s Builder utility followed by minimization in the gas phase using the MMFF94X force field [Molecular Operating Environment (MOE) (2013) Chemical Computing Group Inc., Montreal)]. Structural models of PA3944 WT (PDB ID: 6EDV or 7KPS) and E102A mutant (PDB ID: 7KPP) were prepared in MOE with the Builder utility, then minimized before docking with NANMO. The hydrogen-bonding network of the docking model was further optimized at pH of 7.4 by automatically sampling different tautomer/protomer states using Protonate3D, which calculates optimal protonation states, including titration, rotamer, and “flips” using a large-scale combinatorial search. The active site was specified by the dummy atoms populating the binding pocket. Ligand placement employed the Alpha Triangle method with Affinity dG scoring to generate 1,000 data points per unique ligand that were further refined using the Induced Fit method with GBVI/WSA dG scoring to obtain the top 300 docking poses per ligand. The Amber12:EHT force field was used to perform these calculations. We compared docking using the 6EDV structure with CoA manually acetylated and the 7KPS structure in complex with AcCoA but found no significant differences so we used the higher resolution 6EDV structure for further analyses.

### Multiple Sequence Alignment

The DALI Server was used to identify structures showing the highest similarity to PA3944 (Holm, 2020). The top 11 unique sequences were selected for further analysis. A multiple sequence alignment was performed with the Expresso function on the T-Coffee server (Di Tommaso et al., 2011). The alignment was graphically prepared with ESPript 3.0^1^ (Robert and Gouet, 2014). Each homolog in the alignment is identified by the PDB ID of the structure used to align the sequences. The PA3944 WT protein is designated as PDB ID: 6EDV. Homologs are listed with the following information: PDB ID (Uniprot ID, RMSD to PA3944 in Angstroms, function, and organism, and additional structures of the same protein in the PDB): 3FBU (A0A0F7RDX9, RMSD: 2.1, Uncharacterized, *Bacillus anthracis*); 2FSR (A9CHU9, RMSD: 2.1, Uncharacterized, *Agrobacterium fabrum*); 2ZW7 (Q53796, RMSD: 2.2, Bleomycin Acetyltransferase, *Streptomyces verticillus*; 2ZW4, 2ZW5, 2ZW6, and 2ZW7) (Oda et al., 2010); 3JUW (Q7VZN9, RMSD: 2.2, Uncharacterized, *Bordetella pertussis*); 2FCK (A0A0H3AIE8, RMSD: 2.7, Uncharacterized, *Vibrio cholerae*); 3R96 (Q47510, RMSD: 2.5, Microcin C7 acetyltransferase, *Escherichia coli*; 3R9G, 3R9F, 3R9E, and 3R95) (Agarwal et al., 2011); 6C30 (A0A0D6IYM9, RMSD: 2.4, Uncharacterized, *Mycobacterium smegmatis*; 6C32, 6C37); 1YRE (Q9HYX1, RMSD: 2.4, Uncharacterized, *Pseudomonas aeruginosa*); 1S7N (Q8ZPC0, RMSD: 2.6, RimL, *Salmonella typhimurium;* 1S7L, 1S7K, and 1S7F) (Vetting et al., 2005a); 1NSL (P96579, RMSD: 2.7, Uncharacterized, *Bacillus subtilis*)*;* 2VZZ (P9WQG7, RMSD: 2.7, Uncharacterized, *Mycobacterium tuberculosis;* 2VZZ) (Vetting et al., 2008a).

## Results

### Rationale and Synthesis of Substrate Analog NANMO

Polymyxins are an older class of cationic, macrocyclic cyclic polypeptide antibiotics that have received a renewed interest due to rising antibiotic resistance toward other clinically relevant antibiotics. They are generally produced via bacterial fermentation and therefore exist as mixtures of different structural forms (Falagas and Kasiakou, 2005). Polymyxin B is one member of this family of highly complex macrocyclic structures that contain five diaminobutyric acid (Dab) residues. We previously showed the PA3944 bacterial GNAT enzyme specifically acetylates the 3-Dab residue of polymyxin antibiotics (Czub et al., 2018). This residue is situated in the acyclic portion of the polypeptide between the fatty acid and cyclic peptide of polymyxin antibiotics (Figure 1A). To simplify the characterization of the PA3944 enzyme kinetically without relying on mass spectrometry to measure acetylation of polymyxin B, we designed and synthesized a small molecule mimetic, *N-*(2-aminoethyl)-*N-*methyloctanamide (NANMO, Figure 1A). NANMO is a structural analog of a key portion of polymyxin B that contains both a mimetic of the Dab that is acetylated and a hydrophobic tail. The synthesis of NANMO was accomplished by reacting octanoyl chloride with *N’*-Boc-protected-*N*-methyl-ethylenediamine in the presence of triethylamine in methylene chloride. The Boc group was removed with HCl in diethyl ether providing NANMO as the hydrochloride salt (Figure 1B).

### PA3944 Acetylates NANMO and Polymyxin B With Similar Catalytic Efficiencies

**FIGURE 2:**
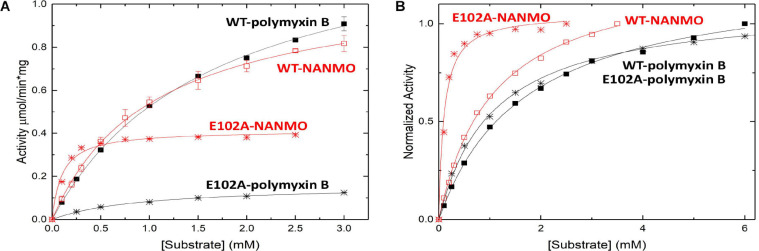
Substrate saturation curves of PA3944 WT and E102A mutant toward polymyxin B and NANMO. The concentration of acceptor substrate was varied while AcCoA was held constant at 0.5 mM. Curves in black correspond to polymyxin B as the substrate and curves in red correspond to NANMO as substrate. WT is shown as solid squares for polymyxin B and open squares for NANMO, E102A is shown as black stars for polymyxin B and red stars for NANMO. **(A)** Substrate saturation curves. **(B)** Normalized data from substrate saturation curves.

We screened the PA3944 enzyme for activity toward NANMO and found it is indeed a substrate (Supplementary Figure S6). Next, we further characterized the WT enzyme toward both NANMO and polymyxin B to compare kinetic parameters. While we characterized this enzyme toward polymyxin B previously (Czub et al., 2018), we chose to recharacterize it alongside the NANMO substrate and PA3944 mutant proteins (discussed below) because polymyxin B is commercially available as a variable mixture. This approach was taken to ensure potential differences or similarities in activity we observed between the two substrates were consistent and not due to different preparations or batches of polymyxin B. When we compared the kinetic parameters of the WT enzyme toward polymyxin B from our previous results and this new preparation, we found the catalytic efficiencies were similar (2.54 × 10^2^ M^–1^s^–1^ from our previous characterization (Czub et al., 2018) compared to 3.0 × 10^2^ M^–1^s^–1^ for this preparation of enzyme and substrate; Table 1). We also found that the WT enzyme used both polymyxin B and NANMO with similar catalytic efficiencies (3.0 × 10^2^ and 3.8 × 10^2^, respectively; Table 1 and Figure 2A), which indicated NANMO could be used as an alternative substrate to characterize the PA3944 enzyme further.

**TABLE 1:** PA3944 wild-type and mutant kinetic parameters toward NANMO and polymyxin B.

Substrate	Enzyme	K_*m*_ (mM)			k_*cat*_ (s^–^^1^)	k_*cat*_/K_*m*_ (M^–^^1^s^–^^1^)
NANMO	WT	1.07	±	0.03	0.41	3.8 × 10^2^
	E102A	0.101	±	0.013	0.15	1.5 × 10^3^
	S148A	N.D.				
Polymyxin B	WT	1.68	±	0.07	0.51	3.0 × 10^2^
	E102A	1.05	±	0.05	0.06	5.7 × 10^1^
	S148A	N.D.				

### S148 Is Critical for PA3944 Catalytic Activity

**FIGURE 3:**
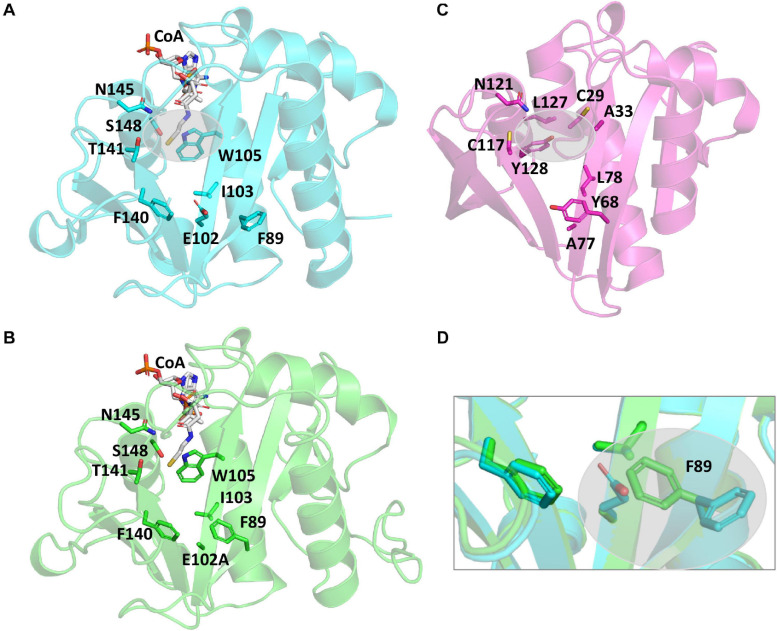
Comparison of PA3944 WT, PA3944 E102A, and PA4794 crystal structures and active sites. **(A)** WT PA3944 structure (cyan; PDB ID 6EDV). CoA is shown with white sticks and key active site residues are shown with cyan sticks. The gray bubble highlights the region of the active site where a general acid residue would typically be located. **(B)** WT PA3944 E102A crystal structure (green; PDB ID 7KPP). CoA is shown with white sticks and key active site residues are shown with green sticks. **(C)** PA4794 crystal structure (pink; PDB ID 5VDB). The ligand was removed for clarity and key active site residues are shown with pink sticks. The gray bubble highlights the region of the active site where a general acid residue would typically be located. **(D)** Overlay of PA3944 WT and E102A structures. The box highlights the F89 residue, which changes conformation when E102 is mutated to alanine. Figures were made using Pymol.

It is well-accepted that GNATs utilize residues in their active sites to accomplish catalysis via a general acid/general base mechanism. However, the PA3944 protein does not contain an obvious general acid, such as a tyrosine residue, in the active site (Figure 3A). Instead, PA3944 has a serine (S148) in a comparable location as the typical critical tyrosine residue found in GNATs. For example, when we compare the active sites of the PA3944 enzyme with the PA4794 enzyme that we previously characterized, we observed there were no other residues in the PA3944 active site that could act as a general acid (compare Figures 3A,C). We compared these two proteins because we had previously shown that a tyrosine (Y128) in the PA4794 enzyme was critical for activity (Majorek et al., 2013). While serine cannot act as a general acid due to its very high pKa (∼16), we considered that it might play a role as a nucleophile in the enzymatic reaction. Therefore, we tested whether S148 was important for kinetic activity by mutating it to alanine and screened the S148A mutant protein toward both polymyxin B and NANMO. We found the S148A mutant was almost completely inactive with activity levels very near the baseline for both of these substrates (not shown). This indicates that S148 is critical for PA3944 enzyme activity.

### PA3944 E102A Point Mutation Affects Enzymatic Activity Differently Depending Upon Substrate

We searched the active site of the PA3944 protein to locate a viable candidate residue that could deprotonate the acceptor substrate. The only residue that could potentially act in this capacity was E102, so we mutated it to alanine and screened the enzyme for activity toward both polymyxin B and NANMO. The E102A enzyme activity toward both substrates decreased compared to WT, with a more significant decrease in activity for polymyxin B compared to NANMO (Figure 2). However, we found the catalytic efficiency of the E102A enzyme varied significantly depending upon the substrate. Specifically, the catalytic efficiency decreased by one order of magnitude compared to WT when polymyxin B was the substrate, but increased by one order of magnitude compared to WT when NANMO was the substrate (Table 1). Thus, when the catalytic efficiencies of the E102A protein toward NANMO and polymyxin B were compared, a substantial difference of two orders of magnitude was observed. This increased catalytic efficiency for the E102A mutant toward NANMO was primarily due to an improved apparent affinity of one order of magnitude compared to WT. On the other hand, the decreased catalytic efficiency for the E102A enzyme for polymyxin B was primarily due to a one order of magnitude decrease in turnover (Table 1 and Figure 2). Based on these results, it appears that E102 more likely plays a role in substrate specificity rather than in the catalytic mechanism.

### Crystal Structure of PA3944 E102A Mutant

Since we observed a significant improvement in catalytic efficiency of the PA3944 E102A enzyme toward NANMO, we crystallized the protein to determine whether we would observe structural changes that might help explain the kinetic observations. The E102A protein crystallized in the same space group (*P*1, with two copies of the monomer in the asymmetric unit) as the WT protein (PDB ID: 6EDV), and no significant deviations to the backbone were observed (Table 2). There were minor perturbations in the positions of the flexible β3-β4 and β6-β7 loops between the mutant and WT protein backbones. The removal of the negative charge from the E102 side chain provided space for the orientation of the phenyl ring of F89 to flip inward compared to the WT structure (Figures 3A,B,D)^2^. Additionally, the removal of the negative charge of E102 increased the overall hydrophobicity of the acceptor substrate binding site. The change in hydrophobicity combined with the conformational change of the F89 residue helps explain the improvement in apparent affinity of the E102A protein for NANMO compared to polymyxin B.

**TABLE 2:** Data collection, structure refinement, and structure quality statistics.

PA3944	WT	E102A
PDB ID	7KPS	7KPP
Diffraction images DOI	10.18430/m37kps	10.18430/m37kpp
Resolution (Å)	50.00–1.80 (1.83–1.80)	50.00–1.45 (1.48–1.45)
Beamline	21-ID-G	19-BM
Wavelength (Å)	0.979	0.979
Space group	*P*1	*P*1
Unit-cell dimensions:	36.6, 44.3, 60.2	36.3, 44.0, 60.2
a, b, c (Å)		
Angles: α, β, γ (^*o*^)	97.9, 106.7, 89.9	98.0 106.9, 90.0
Protein chains in the ASU	2	2
Completeness (%)	97.1 (95.6)	96.3 (93.1)
Number of unique reflections	32,365	60,303
Redundancy	2.2 (1.9)	4.7 (4.0)
< | > / <σ(I)>	10.0 (1.4)	31.9 (5.9)
CC½	0.55	0.94
R_*merge*_	0.095 (0.598)	0.053 (0.210)
R_*work*_/R_*free*_	0.206 / 0.235	0.149/0.168
Bond lengths RMSD (Å)	0.003	0.004
Bond angles RMSD (°)	1.2	1.3
Mean B value (Å^2^)	25	17
Number of protein atoms	2,995	3,097
Mean B value for protein (Å^2^)	24	15
Number of water molecules	287	588
Mean B value for water molecules (Å^2^)	32	29
Clashscore	2.60	1.10
MolProbity score	1.05	0.82
Rotamer outliers (%)	0.0	0.00
Ramachandran outliers (%)	0.0	0.00
Ramachandran favored (%)	98.3	98.6

*Values in parentheses are for the highest resolution shell. Ramachandran plot statistics are calculated by MolProbity.*

### Conservation of S148 and E102 in Homologs

**FIGURE 4:**
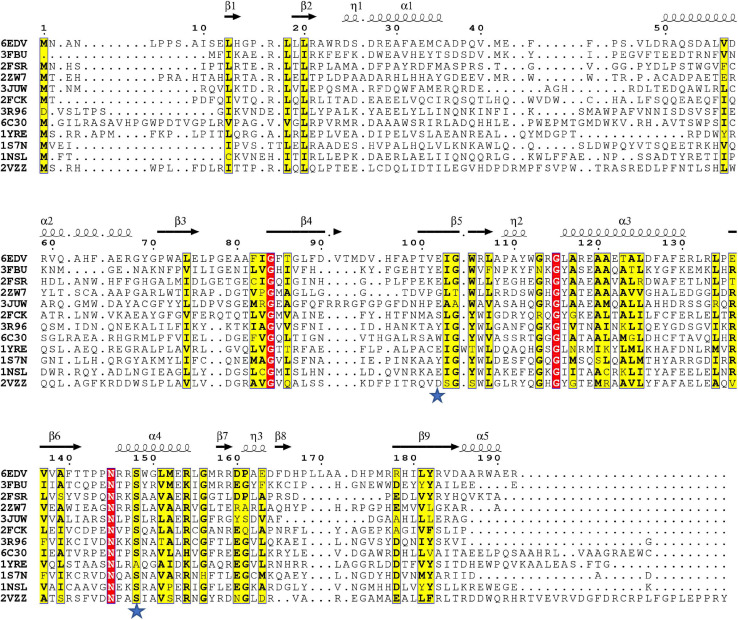
Structure-based multiple sequence alignment of PA3944 and homologs. Residues boxed in red indicate strict conservation, while residues boxed in yellow indicate greater than or equal to 70% identity across the 11 homologs. Each homolog is identified by the PDB ID of the structure used to help align the sequences and further information about the identities of specific proteins is located in section “Materials and Methods.” Blue stars indicate location of PA3944 E102 and S148 residues characterized in this study. The figure was made using ESPRIPT.

Since the S148 residue was critical for activity, we searched for homologs of the PA3944 enzyme with the objective of determining whether this residue is conserved. To identify proteins showing the highest structural similarity, we submitted the PA3944 monomer (PDB ID: 6EDV) to the DALI server (Holm, 2020). Eleven unique GNAT structures with the highest structural alignment scores were selected for further sequence analysis. These structures exhibited RMSDs ranging from 2.1 to 2.7 Å, while their sequence identities ranged from 11 to 30%. This high structural similarity but low sequence identity is typical within the functionally diverse GNAT superfamily. We performed a structure-based sequence alignment to better align the active site residues between the analyzed proteins. We found that S148 is nearly 100% conserved (10 of the 11 closest homologs have this residue) (Figure 4). The single homolog that did not have this serine conserved was the uncharacterized PA3270 (PDB ID: 1YRE) protein, which instead has an alanine residue in this position. On the other hand, E102 is less well conserved among homologs, with only 6 of the 11 closest homologs having the corresponding E102 residue.

### Mapping Conserved Residues Onto the PA3944 Structure

**FIGURE 5:**
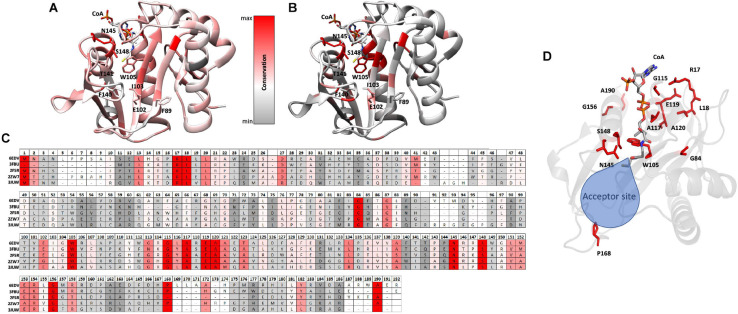
Conserved residues of homologs mapped onto the PA3944 WT structure (PDB ID: 6EDV). **(A)** Conserved residues of all 11 homologs identified by DALI mapped onto the PA3944 WT structure. **(B)** Conserved residues of top four homologs (PDB IDs: 3FBU, 2FSR, 2ZW7, and 3JUW) mapped onto the PA3944 WT structure. Residues are colored in a step gradient from gray (no to low conservation) to red (high conservation) and CoA is present in the acyl donor site of the PA3944 structure. Key residues in the donor and acceptor sites are shown in sticks. **(C)** Linear sequence comparison of top 4 homologs and PA3944 colored by conservation. **(D)** Rotated view of PA3944 with acceptor site indicated and all red (completely conserved) residues and CoA shown as sticks. The majority of the conserved residues are found in the donor site or near the catalytic center where the acyl donor site and acceptor sites join.

To examine the conservation of additional residues across homologs from our DALI search, we performed the following experiment. First, we input our T-Coffee multiple sequence alignment file into the Chimera Al2CO program to generate scores reflecting sequence conservation for all 11 homologs vs. the top 4 homologs. These scores were then color-coded based on numerical thresholds and mapped onto the PA3944 structure to visualize the locations in 3D of conserved residues (Figures 5A,B). We selected the top four homologs [3FBU (2.1 Å RMSD, 28% identity), 2FSR (2.1 Å RMSD, 30% identity), 2ZW7 (2.3 Å RMSD, 23% identity), and 3JUW (2.2 Å RMSD, 24% identity)] for comparison based on highest sequence identity and best RMSD.

When we compared the residue conservation between all 11 homologs, we observed a core set of residues present in the top 4 that were much more restricted. By splitting the analysis into two separate comparisons (4 vs. 11 homologs), different trends for conserved residues were revealed and provide additional information as to which residues may be required for more specific substrate recognition. For example, when all 11 homolog sequences are mapped onto the structure, the conservation is more broadly distributed across the entire sequence and mirrors the sequence alignment shown in Figure 4 wherein the β4/β5 strands and the AcCoA binding site are generally the most well-conserved. Interestingly, there is a stark contrast in some of the residues that are conserved in the top 4 homologs compared to all 11 homologs. For example, W105 is conserved in the top 4 homologs but is substituted as Y, T, or S in other homologs. To further explore the identities of these core residues in the top 4 homologs, we mapped the scores and color coding to the primary sequence (Figure 5C) and generated a figure highlighting these residues in 3D (Figure 5D). Based on this analysis, we found most of the conserved residues are located in or near the AcCoA binding site except for P168. Therefore, it is highly likely these top 4 homologs have a different function than PA3944. However, all of the homologs have S148 conserved, indicating they likely use a similar catalytic mechanism.

### PA3944 Uses a Ping-Pong or Hybrid Ping-Pong Kinetic Mechanism Depending on Substrate

**FIGURE 6:**
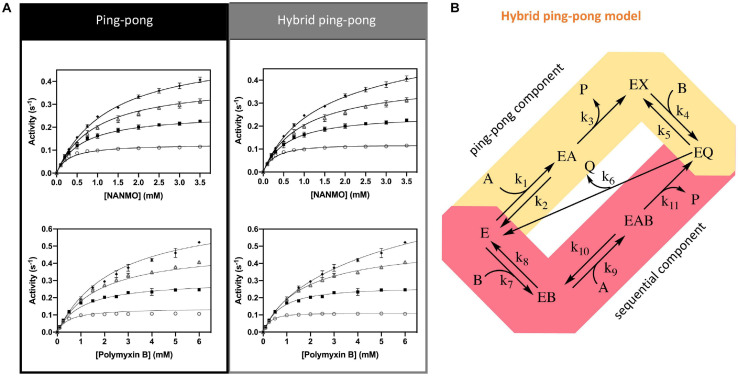
Kinetic mechanism and fitting of kinetic data to two kinetic models. **(A)** Ping-pong and hybrid ping-pong kinetic models fitted to a series of kinetic curves toward acetylation of NANMO or polymyxin B at varying concentrations of AcCoA. **(B)** Hybrid ping-pong model with two components: a ping-pong path and a sequential path. E is enzyme, EX is acetylated enzyme, A is AcCoA, B is acceptor substrate (polymyxin B or NANMO), P is CoA, and Q is acetylated acceptor product. The hybrid model allows free enzyme to bind AcCoA or acceptor substrate at the same time. If it binds AcCoA first, the enzyme becomes acetylated and the ping-pong path is used, whereas if acceptor substrate binds first the acetyl group of AcCoA can be transferred directly to acceptor substrate using the sequential path (see section “Materials and Methods” for more details).

Since we found S148 is critical for enzyme activity, and there is no viable residue that could potentially act as a general acid, we suspected S148 may act as a nucleophile in the chemical reaction. Therefore, we sought to determine the PA3944 enzyme kinetic mechanism. We performed steady-state enzymatic assays to generate a series of substrate saturation curves toward both NANMO and polymyxin B (acceptors) against four different concentrations of AcCoA (donor). These families of curves were then fitted to a series of kinetic models as described previously (Filippova et al., 2015). Since NANMO was the simpler substrate, we first fitted the kinetic data to the classical ordered, random, and ping-pong kinetic models. The model with the best fit to the NANMO data was a ping-pong kinetic mechanism (Figure 6A, Table 3, and Supplementary Figure S5). In this model, a covalent enzyme intermediate is implicated. AcCoA first binds to the enzyme and an acyl-enzyme intermediate must be formed using a nucleophilic amino acid in the active site. Then, the acyl group is transferred from the acylated enzyme residue to the second substrate (Figure 6B).

**TABLE 3:** Analysis of fitting for kinetic mechanism models toward both NANMO and polymyxin B.

Acceptor substrate	Model	AICc	ΔAICc	Relative likelihood
NANMO	Random	−406	90	2.86 × 10^–20^
	Ordered AB	−341	155	2.20 × 10^–34^
	Ordered BA	−338	158	4.91 × 10^–35^
	**Ping-pong**	−**495**	**1**	**0.607**
	**Hybrid**	−**496**	**0**	**1**
Polymyxin B	Random	−360	135	4.84 × 10^–30^
	Ordered AB	−311	184	1.11 × 10^–40^
	Ordered BA	−311	184	1.11 × 10^–40^
	Ping-pong	−411	84	5.75 × 10^–19^
	**Hybrid**	−**495**	**0**	**1**

*Each model is defined in the section “Materials and Methods” and the derivation of the equation for the hybrid ping-pong model is located in Supplementary Scheme S1. The models that best fit the data for each substrate are bolded. Since the relative likelihood values for the ping-pong and hybrid ping-pong models toward NANMO were quite similar, we could not discard either model; the hybrid model could be slightly preferred. Therefore, both models are bolded. The corrected Akaike Information Criterion (AICc) obtained from fitting various models to the enzyme kinetic data is shown. The difference of two AICc values for each model compared to the one with the lowest value is shown as ΔAICc. The relative likelihood was calculated using the equation *e*^−0.5(△*AIC*_*c*_)^.*

Next, we fitted the kinetic data obtained toward polymyxin B to the same set of traditional kinetic models as for NANMO. However, none of these models were sufficient to explain the enzyme behavior. When we compared data fitting to the ping-pong model, the curves were biased and the standard deviation was high (0.013 s^–1^); therefore, we explored alternative models. A relatively simple hybrid ping-pong model exhibited the lowest AICc value, eliminated the bias from the fitting and had a lower standard deviation (0.005 s^–1^) (Figures 6A,B, Table 3, and Supplementary Figure S5). This is a simplified hybrid ping-pong mechanism from one described before (Segel, 1975) and it implies the enzyme is able to bind both donor and acceptor at the same time. Our hybrid model contains two major paths: one is the classical ping-pong reaction and the second is the classical sequential scheme where AcCoA binds first to the enzyme and the acyl group is transferred to the second substrate (Figure 6B).

Based on these results, we then fitted the data obtained when NANMO was the substrate to the hybrid model. Both ping-pong and hybrid models produced nearly identical AICc and relative likelihood values, which indicates the ping-pong model is sufficient to explain the NANMO data (Table 3). A more complex hybrid model does not contribute to a more advantageous fit but both are possible and we cannot exclude either model based on the AICc values. It is possible that a fraction of the reaction can proceed through the sequential path of the hybrid model when NANMO is the substrate, but the rate constant for this path must be significantly lower than for the ping-pong path. On the other hand, when polymyxin B is the substrate, the rate constants for the two paths may not deviate as drastically, and therefore a larger fraction of the reaction compared to NANMO may proceed through the sequential path. The enzyme may exhibit this variability when polymyxin B is the substrate because it is a larger molecule than NANMO and is likely to remain in the active site longer. Thus, the substrate is directing the preferred path for acetylation, but in all cases there is an underlying ping-pong component present. This indicates the role of serine as a nucleophile is plausible and it must become acylated during the reaction. Even if a fraction of the reaction occurs via the sequential path, we cannot discount the possibility that S148 receives the acyl group from AcCoA and immediately transfers it to the acceptor substrate.

### No Acetylated S148 Observed in the PA3944 Crystal Structure

Since our kinetic studies showed the enzyme utilized a ping-pong or hybrid ping-pong mechanism, we attempted to obtain a crystal structure of the protein with S148 acetylated. All of our previous structures of the enzyme were determined in the presence of CoA. Therefore, we co-crystallized the protein in the presence of AcCoA and looked for density around S148 for an acetyl group. In addition to adding AcCoA for co-crystallization, we also included (R)-3-(2-chloroacetamido)-4-(((S)-1-methoxy-1-oxo-3-phenylpropan-2-yl)amino)-4-oxobutanoic acid. While this compound was present in the crystallization solution, it was not observed in the crystal structure; the rationale and synthesis of this compound as an alternate substrate will be described in a separate manuscript. In the presence of these compounds, the enzyme also crystallized in the *P*1 space group with two molecules in the asymmetric unit (Table 2). Based on the observed electron density, we modeled CoA in chain A and AcCoA in chain B. In this structure, we did not observe any additional density around S148 that would indicate its acetylation in the crystal. However, we did observe a very small amount of AcCoA cleavage in the presence of the enzyme and absence of acceptor substrates in our kinetic assays, possibly indicating the enzyme could form an acyl-enzyme intermediate required for a kinetic mechanism with a ping-pong component (Supplementary Figure S6). Further studies are necessary to show the acyl-enzyme intermediate is indeed formed but are beyond the scope of this current study. This structure was nonetheless useful for docking experiments.

### NANMO Docking Into PA3944 WT and E102A Crystal Structures

Since we did not have a structure of the PA3944 WT or E102A proteins in complex with NANMO, and did not observe density for an acetylated S148 residue, we used molecular docking as a tool for addressing questions about how NANMO might bind and how the enzyme catalyzes its reaction. We chose to focus on NANMO due to its chemical homogeneity and simpler structure than polymyxin B. Therefore, NANMO was docked into both WT and E102A structures using the following framework (Supplementary Figures S7, S8). We compared results of docking NANMO in the protonated form (NANMO⋅H^+^) and deprotonated form (NANMO free base) in structures with S148 either non-acetylated or acetylated (Ac-S148) and in the presence of AcCoA or CoA, respectively. Our docking experiments showed NANMO bound to the acceptor site of both the WT and E102A structures when either AcCoA or CoA was present and when S148 was acetylated or not acetylated. When we docked NANMO into the structure without AcCoA/CoA, the ligand bound to the AcCoA/CoA donor site of the protein (not shown). Therefore, the remainder of our experiments contained either AcCoA or CoA in the donor site, which is compatible with the hybrid kinetic model in which both acceptor and donor can bind to the enzyme at the same time. Moreover, the docking studies indicate S148 can be acetylated and still bind NANMO in the acceptor site in a reasonable location for acetyl transfer to occur.

In order for the conjugate acid of the primary amine of an acceptor substrate to become acetylated, it must first be deprotonated. Therefore, we first docked NANMO into the WT structure with AcCoA to determine which residues might interact with the protonated and free base forms of the molecule. We selected representative docking poses with the lowest docking scores and analyzed the binding orientations and interactions of NANMO (Supplementary Figures S7, S8). When NANMO was protonated, its terminal amine formed an H-bond with E102 and with the carbonyl oxygen of AcCoA. NANMO also exhibited stabilizing interactions with F44, F140, H167, and H179. In contrast, when the free base was docked, the carbonyl oxygen of NANMO formed an H-bond with H167 and the terminal amine formed an H-bond with the carbonyl oxygen of AcCoA. Ligand stabilizing interactions also occurred with F44, F89, F140, and E102. When we compared these results with the docking of protonated and free base forms of NANMO into the WT structure with S148 acetylated (S148-Ac) and CoA, we found the terminal amine of protonated NANMO formed H-bonds with the side chain of E102 and the backbone oxygens of I103 and F140. Significant stabilization of the amino-methyl group of NANMO occurred with F44. In the free base form, NANMO maintained H-bonding interactions with E102 and the backbone oxygen of I103.

When we examined the same docking studies but in the PA3944 E102A structure, we found the protonated NANMO in presence of AcCoA formed an H-bond with the backbone oxygen of F140 but the free base NANMO formed an H-bond with the backbone oxygen of F43. When S148 was acetylated the terminal amine of protonated NANMO formed H-bonds with the backbone oxygens of I103 and F140 and carbonyl oxygen of Ac-Ser148. The NANMO free base in the E102A structure formed H-bonds with the backbone oxygen of I103 and the carbonyl oxygen of Ac-Ser148. Therefore, it appears backbone oxygens of I103 and/or F140 are critical for H-bonding to the NANMO substrate when S148 is acetylated regardless of NANMO protonation state in either WT or E102A enzymes. No clear pattern emerged when S148 was not acetylated and the protonation state of NANMO did not appear to drastically alter its interactions in either WT or E102A structures.

### The Acceptor Site of PA3944 Is Quite Large Compared to the Size of NANMO

**FIGURE 7:**
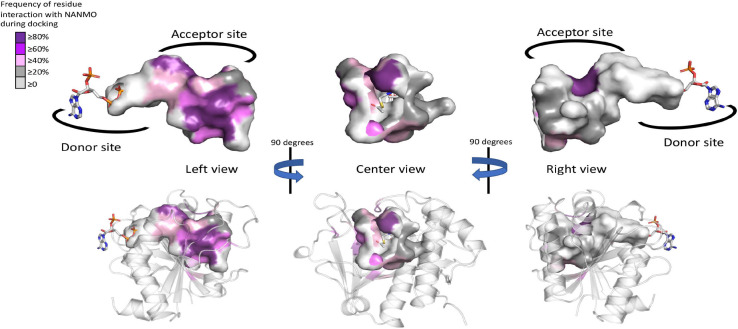
Frequency of WT PA3944 acceptor site interactions with NANMO during docking studies. All ligand interaction maps across all docking experiments with NANMO pointed toward the donor site were compiled and were analyzed to determine residues that most frequently interacted with NANMO. Residues were colored based on frequency (gradient from purple for high frequency to gray for low frequency) on the surface of the acceptor site. AcCoA is shown as sticks. More specific details of separate docking studies can be found in Supplementary Table S1.

The WT PA3944 acceptor site of the PA3944 enzyme contains predominantly non-polar residues and only a few polar residues, and its acceptor binding pocket is large enough to accommodate multiple conformations of the NANMO ligand. Therefore, we sought to determine which residues of the acceptor site more frequently interacted with the NANMO ligand across 100 of the lowest energy poses by analyzing the interaction maps generated for each of these poses. We selected poses for further analysis using the following criteria: NANMO needed to interact with acceptor site residues and have the terminal amine pointed toward the donor site. We found the residues that were in close proximity to NANMO across WT docking studies included: F43, F44, P45, L56, R59, P71, F85, F89, M93, E102, I103, G104, R106, F140, T141, T142, N145, S148, H167, L169, L170, M176, and H179. When we docked NANMO into the E102A structure, we found two additional residues that were located in close proximity to the molecule that were not observed in the WT docking: W105 and M152 (Figure 7 and Supplementary Table S1). The residues with the highest frequency of interaction (>50% average across all perturbations) with NANMO included F43, F44, P45, F89, E102, F140, T141, T142, H167, and H179 (Supplementary Table S1) and were primarily localized on one side of the acceptor pocket, with the exception of F44 and F45, which reside at the top of the pocket near the active center (Figure 7).

## Discussion

### PA3944 Does Not Use a General Acid/General Base Chemical Mechanism

Based on the crystal structures and kinetic data of PA3944 we have presented, it is clear that PA3944 does not proceed through a general acid/base mechanism as most other characterized GNATs. Our results provide multiple lines of evidence that support this conclusion. First, the PA3944 enzyme has a serine residue (S148) in place of the oft-conserved general acid tyrosine. Here, we showed this residue is critical for catalysis when either NANMO or polymyxin B are substrates. It has been suggested that serine can act as a general acid in other GNATs (O’Flynn et al., 2018), but its high pKa makes it highly unlikely to act in this capacity. Based on the crystal structure of PA3944, there are no other residues in the active site that could act as a general acid and the only viably positioned residue that could act as a general base is E102. However, when we mutated this residue we showed it is not required for catalysis. Indeed, the differential responses to polymyxin B vs. NANMO and variable conservation of this residue in an otherwise well-conserved region suggests that it more likely plays a role in substrate recruitment or binding. This type of behavior has also been observed for the E72 residue of the aminoglycoside acetyltransferase AAC(6′)-Ii, where it was shown to be critical for activity toward aminoglycosides but not the poly-L-lysine peptide substrate and did not act as a general base (Draker and Wright, 2004). While it is not unprecedented in the GNAT superfamily for oligomerization to play a role in providing residues from different protomers to construct the active site (Hegde et al., 2007; Richter et al., 2020), we previously showed that the PA3944 enzyme is a monomer in solution (Czub et al., 2018). At least under our described *in vitro* assay conditions and with the substrates we tested, there appear to be no additional residues that could serve the role of general acid or general base. Therefore, an alternative chemical mechanism is likely for this enzyme and others that share a similar active site composition.

### Critical Residues and Kinetic Studies of PA3944 Homologs

Of the 11 homologs with structures we selected for analysis, only 3 have been kinetically characterized. These include the protein N-terminal acetyltransferase RimL (PDB ID: 1S7N) from *Salmonella typhimurium*, the MccE Microcin C7 acetyltransferase from *E. coli* (PDB ID: 3R96), and the bleomycin acetyltransferase from *Streptomyces verticillus* (PDB ID: 2ZW7). Studies of the RimL protein showed an active site cysteine residue was not critical for activity and instead suggested S141 (equivalent to S148 in PA3944) might act as a general acid and E160 might act as a general base in the reaction; however, kinetic studies to test this were not presented (Vetting et al., 2005a). The MccE study suggested S553 (equivalent to S148 in PA3944) and E572 (no equivalent residue in PA3944) were positioned to act as the general acid and base in the enzymatic reaction. The activity of the S553/E572 double mutant *in vivo* exhibited a 25-fold decrease in activity, which indicated these residues were critical for activity (Novikova et al., 2010). Single mutant studies to determine whether both residues were required for this reduction in activity were not reported. Kinetic studies of the bleomycin acetyltransferase suggest that the enzyme forms a ternary complex and that the product release is ordered with CoA leaving last; the binding order of bleomycin and AcCoA was suggested to be flexible. Ultimately, residues that could act as a general acid or base were not identified. Instead, it was suggested that the non-polar residues within a tunnel in the active site decrease the pKa of the amine of bleomycin upon approach to the active site (Oda et al., 2010). Furthermore, nearly all analyzed homologs with structures (10 of 11) had a serine at the corresponding position of S148 in PA3944, suggesting that the role of this residue is highly conserved. However, the presence of S148 is not an indicator of substrate preference as several enzymes with a conserved serine residue acetylate a variety of substrates, including peptidic/amino acids [MccE: processed Microcin C (Novikova et al., 2010; Agarwal et al., 2011), BmNat: Bleomycin (Oda et al., 2010); RimL: N-terminal amines (Vetting et al., 2005a), EctA: free Dab (Richter et al., 2020)], aminoglycosides [AAC(6′)-Iy (Vetting et al., 2004)], arylalkylamines [AANAT7 (Han et al., 2012)], and polyamines [vPat (Charlop-Powers et al., 2012)].

### Canonical and Divergent Chemical Mechanisms in GNATs

Despite the structural and functional diversity of GNAT superfamily members, the catalytic and kinetic mechanisms of GNATs in the majority of literature reports are largely presumed to be well-established and nearly uniform. The canonical catalytic mechanism for GNATs is a general acid/base-catalyzed mechanism, while the kinetic mechanism is a direct acetyl-transfer mechanism (Siehl et al., 2007; Vetting et al., 2008b; Favrot et al., 2016; Burckhardt and Escalante-Semerena, 2020). Contrary to this perspective, several members of the GNAT superfamily actually utilize a range of catalytic mechanisms. For example, substrate-assisted catalysis has been proposed for some GNATs whereby the CoA thiolate or CoA adenine participates in the catalytic mechanism (Farazi et al., 2001; O’Flynn et al., 2018). More intricate residue interactions have also been suggested in the cases of aminoglycoside acetyltransferase AAC(3′)-VIa and dopamine acetyltransferase (DAT). The AAC(3′)-VIa protein may use a non-canonical catalytic triad stabilized by a low barrier hydrogen bond involving glutamate, histidine, and the substrate (Kumar et al., 2018), whereas DAT may use a serine-serine-glutamate catalytic triad (Cheng et al., 2012). Moreover, the aminoglycoside 6′-*N*-acetyltransferase AAC(6′)-Ii enzyme does not use a general acid/base mechanism even though it has conserved residues compared to homologs that use these residues for catalysis. Instead, these residues are proposed to bind and orient substrates for catalysis (Draker and Wright, 2004). Finally, in absence of a nearby viable acid or base, a proton wire has been proposed for several GNATs (Hickman et al., 1999; Vetting et al., 2002; Montemayor and Hoffman, 2008). This wire can allow the general base (or acid) residue to be located more distally from the active center, and abstracts or donates a proton through a network of residues and/or ordered water molecules (Brent et al., 2009; Charlop-Powers et al., 2012). The CoA has been proposed to be reprotonated by the acceptor amine upon collapse of the tetrahedral intermediate in absence of a general acid (Dempsey et al., 2014). Here, we have provided evidence that PA3944 and its homologs represent an additional deviation from the canonical chemical mechanism for GNATs.

### Serine as a Nucleophile in GNAT Enzymatic Reactions: A New Paradigm for GNAT Chemical Mechanisms

**FIGURE 8:**
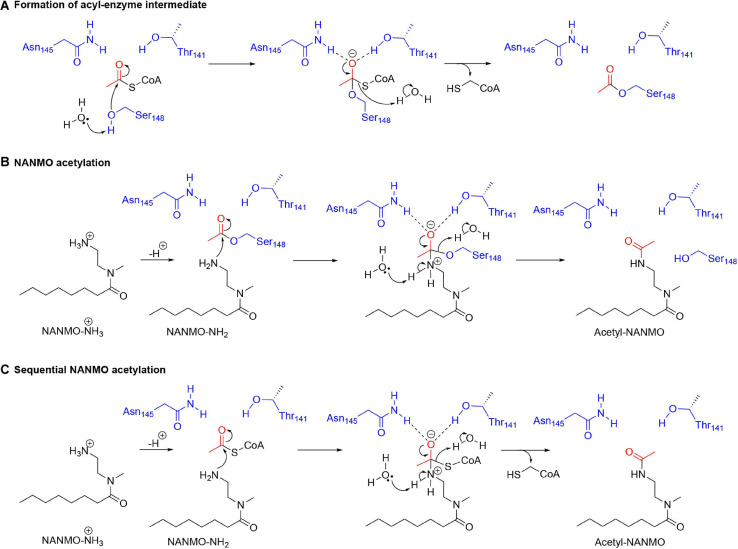
Proposed chemical mechanism for PA3944 using both ping-pong and sequential components of the hybrid mechanism. In the ping-pong component, **(A)** S148 acts as a nucleophile and is acetylated during the enzymatic reaction and then **(B)** deprotonated NANMO is acetylated by the acyl enzyme. In the sequential component, **(C)** direct enzyme-mediated acetylation of NANMO by AcCoA occurs.

Our kinetic data for the PA3944 enzyme demonstrate that of the models we tested, the hybrid ping-pong kinetic mechanism, which contains both ping-pong and sequential components, explains data collected for both polymyxin B and NANMO acceptor substrates. Hybrid ping-pong mechanisms arise when there is a covalently modified enzyme intermediate in an enzyme with separate substrate binding sites (Northrop, 1969; Wong and Wong, 1983). Since this mechanism requires formation of an acyl-enzyme intermediate, it must utilize a nucleophilic amino acid during the reaction. Here, we have provided evidence that S148 plays the role of an active site nucleophile that enables the ping-pong component of the hybrid mechanism to proceed. Based on our *in vitro* and *in silico* experiments, we propose the following chemical mechanism for the PA3944 ping-pong component (Figures 8A,B). We used NANMO as a representative substrate, but the mechanism applies to polymyxin B as well. First, AcCoA binds to the donor site of PA3944 and S148 is likely deprotonated by a water molecule concomitant with a nucleophilic attack on the carbonyl carbon of AcCoA. The tetrahedral intermediate is stabilized by an oxyanion hole formed by the side chain amide of N145 and the side chain hydroxyl of T141. A water molecule likely facilitates the collapse of this complex and releases CoA to form the acetylated S148-enzyme intermediate. Next, NANMO is either deprotonated upon approach of the substrate into the active site through an unidentified base or is already deprotonated due to its relatively low pKa. NANMO then attacks the carbonyl carbon on the acetylated S148 residue and forms a second tetrahedral complex, again stabilized by a potential oxyanion hole with the side chain amide of N145 and the hydroxyl of T141. The protonation of the oxygen atom on S148 and delocalization of the electrons from the oxyanion releases the acetylated NANMO product and enables S148 to be restored for another round of catalysis when a new molecule of AcCoA binds. As there is no residue suitably placed for deprotonation of S148 we believe that a network of water molecules likely facilitates this process. For the sequential component of the hybrid mechanism (Figure 8C) to proceed, the free base of NANMO directly attacks AcCoA, providing the tetrahedral intermediate wherein the alkoxide moiety is stabilized by the oxyanion hole formed by Asn145 and Thr141, and possibly an additional water molecule. Collapse of the tetrahedral intermediate with the expulsion of CoA and proton transfers via a network of water molecules in the aqueous environment then provides the acetylated NANMO product.

### Kinetic Mechanisms of Enzymes in the GNAT Superfamily

To our knowledge, only a single GNAT (indolamine *N-*acetytransferase from *Periplaneta americana*; PaNAT) has previously been demonstrated to employ a ping-pong mechanism (Sakamoto et al., 1998). However, the enzyme was not purified to homogeneity and has largely been ignored in the literature and in follow-up experiments. Nevertheless, S205 in PaNAT appears equivalent to S148 in PA3944 and could conceivably act as a nucleophile. It should be noted that this serine is strictly conserved in the iAANATs as well but these enzymes utilize a proton wire catalytic mechanism (O’Flynn et al., 2018). Therefore, the role of the conserved serine residue in catalysis is not pre-ordained strictly by conservation. Of the GNATs that have had their kinetic mechanism experimentally determined, these enzymes overwhelmingly favor a direct transfer/sequential mechanism regardless of the identity of their acceptor substrate classification. Indeed, a large number of kinetic studies have shown that Gcn5/pCAF histone *N-*acetyltransferases (HATs) (Jiang et al., 2012), arylalkylamine *N-*acetyltransferases (AANATs) (Dempsey et al., 2014; Battistini et al., 2019), aminoglycoside *N-*acetyltransferases (AACs) (Vetting et al., 2008b), spermidine/spermine *N-*acetyltransferases (SSATs) (Bewley et al., 2006; Filippova et al., 2015), and GNATs with unknown native substrates (Majorek et al., 2017; Reidl et al., 2017) all utilize some form of a direct transfer mechanism.

### Non-GNAT Acetyltransferases That Utilize Ping-Pong Kinetic Mechanisms

While ping-pong mechanisms are rarely identified in the GNAT superfamily literature, many other enzymes in the diverse array of acetyltransferase families have been characterized to employ such mechanisms. In our experience, the literature on the different types of acetyltransferase families can be conflicting, so sometimes kinetic mechanisms for non-GNAT enzymes are incorrectly attributed to GNAT enzymes. Several examples of acetyltransferases in non-GNAT families that were originally thought to utilize a ping-pong mechanism but were later shown to utilize other kinetic mechanisms include the Esa1 acetyltransferase (MYST family) (Yan et al., 2002) and p300 acetyltransferase (Thompson et al., 2001). In the case of Esa1, the hypothesized acceptor cysteine was shown to be unimportant for catalysis and the kinetics adhere to a direct transfer mechanism (Berndsen et al., 2007). In the case of p300, the hypothesized acceptor cysteine was unreactive with AcCoA (Hwang et al., 2007) and the enzyme was subsequently shown to proceed through an unusual Theorell-Chance mechanism (Liu et al., 2008).

Examples of non-GNAT enzymes with clearly demonstrated ping-pong mechanisms include the following. The H4 histone acetyltransferase (Kat8; MYST family) uses a ping-pong mechanism to acetylate the Nε of K16 on the H4 histone. Based on the crystal structure, it was hypothesized that C143 acts as the acetyl-acceptor, but no mutagenesis was performed (Wapenaar et al., 2015). It appears the majority of acetyltransferases that use AcCoA as the donor and perform ping-pong mechanisms have a conserved catalytic triad. The YopJ effector family is a class of protein *N*-acetyltransferases that features a catalytic triad (Glu/His/Cys or Asp/His/Cys) that is homologous to the ubiquitin-like proteases. YopJ was initially hypothesized to employ a ping-pong mechanism based on its similarity to cysteine proteases (Mukherjee et al., 2007), and the structural characterization of the acetyl-cysteine intermediate supported this result (Zhang et al., 2017). The arylamine *N*-acetyltransferase family (not to be confused with the GNAT arylalkylamine *N*-acetyltransferases) exists in both prokaryotes [such as *M. tuberculosis* TBNAT (Sikora et al., 2008)] and eukaryotes [such as hamster NAT2 (Wang et al., 2005)] and utilizes a strictly conserved catalytic triad (Glu/His/Cys or Asp/His/Cys) and an ordered bi-bi ping-pong mechanism (Westwood et al., 2006; Kubiak et al., 2013; Zhou et al., 2013). Peptidoglycan *O*-acetyltransferases (OatA and OatC) also proceed through a bi-bi ping-pong mechanism with a conserved catalytic triad (Asp/His/Ser)(Bergfeld et al., 2009; Sychantha and Clarke, 2018). Given the intricacies involved with various acetyltransferase studies, it is clear the kinetic mechanisms for this large class of enzymes may be more complex than originally thought. Therefore, the knowledge of these enzyme mechanisms is currently in a fluid state where new information is still impacting our understanding of how acetyltransferases from multiple families function.

### Evolution of GNAT Kinetic and Chemical Mechanisms

**FIGURE 9:**
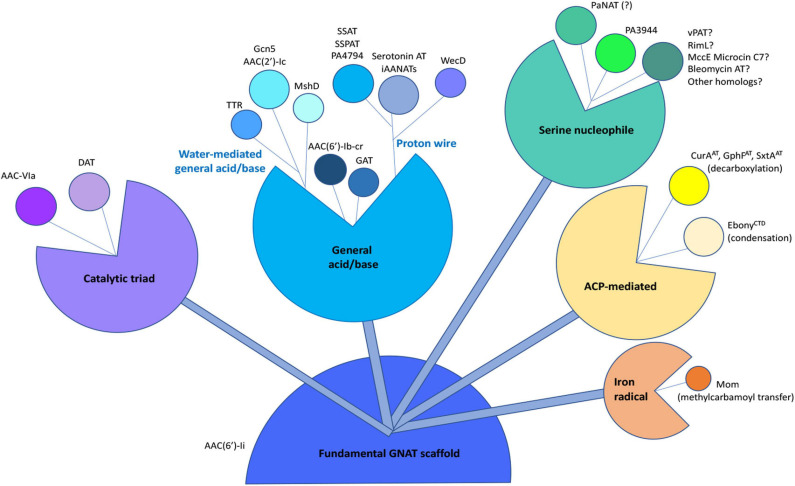
Compilation of known chemical mechanisms of GNATs in the primary literature. All GNATs evolved from a common scaffold, which later gave rise to a variety of chemical mechanisms, including the general acid/base, proton wire (or remote general base or catalytic water), catalytic triad, serine nucleophile, acyl-carrier protein (ACP)-mediated, or iron radical-mediated. Examples of each type of chemical mechanism are indicated adjacent to each bud within the different sub-classes. The PA3944 enzyme from the current study is shown in bright green. Details of abbreviations and corresponding references for each example listed can be found in Supplementary Table S2.

It has been previously proposed that the GNAT fold acts as a scaffold to enable the acyl-donor and acceptor substrates to bind in a proper orientation for catalysis to occur (Dyda et al., 2000; Draker and Wright, 2004). Draker and Wright (2004) also suggested the acyl-acceptor molecule may dictate differences in transfer chemistry. Based on many studies of the chemical and kinetic mechanisms of GNAT enzymes, it is clear this superfamily has evolved to utilize a diversity of chemistries and active site residues for more complex and targeted modifications of acceptor substrates. Identification of additional non-acyl transfer reactions for GNATs, including decarboxylation, methylcarbamoyl transfer, and condensation (Gu et al., 2007; Izoré et al., 2019; Karambelkar et al., 2020; Skiba et al., 2020) highlight the shear multitude of chemistries that enzymes from this superfamily can accomplish. Thus, GNATs appear to have a highly tunable scaffold that has evolved to modify a diverse range of substrates. Based on the current state of knowledge in this field, we present a compilation of some GNAT chemistries derived from a fundamental GNAT scaffold (Figure 9). Our results with PA3944 show yet another divergence to the canonical chemical and kinetic mechanisms for this family of proteins. Thus, as our structural, functional, and mechanistic knowledge of these enzymes increases, some of our former broad generalizations about how GNATs catalyze their reactions should be reassessed.

## Data Availability Statement

The datasets generated for this study can be found in the online repositories. The names of the repository/repositories and accession number(s) can be found in the article/Supplementary Material.

## Author Contributions

JB and MK wrote the first draft of the manuscript. KM, DB, and MK conceptualized the study. DB, MB, and MK were integral to kinetic and/or chemical mechanism data interpretation. JB, TH, MC, KM, XA, WM, MB, DB, and MK edited the final draft manuscript. All authors performed the experiments and/or data analysis.

## Conflict of Interest

The authors declare that the research was conducted in the absence of any commercial or financial relationships that could be construed as a potential conflict of interest.

## Footnotes

1 http://espript.ibcp.fr

2 https://molstack.bioreproducibility.org/project/view/w7qpz5GeCt6pr9ikpoDi/
