# Supplementary material for: Gcn5-Related N-Acetyltransferases (GNATs) With a Catalytic Serine Residue Can Play Ping-Pong Too
Source: Front Mol Biosci. 2021 Apr 12;8:646046. doi: 10.3389/fmolb.2021.646046 (PMC8072286; doi:10.3389/fmolb.2021.646046)
Supplement: Supplementary file 1 [file Data_Sheet_1.docx]

**Supplemental Materials**

**Supplemental Figures and Tables**

|  | **Content** | **Page** |
| --- | --- | --- |
| **Figure S1** | *N*-(2-Aminoethyl)-*N*-methyloctanamide hydrochloride (NANMO) ^1^H NMR | 2 |
| **Figure S2** | *N*-(2-Aminoethyl)-*N*-methyloctanamide hydrochloride (NANMO) ^1^H NMR expansion | 3 |
| **Figure S3** | *N*-(2-Aminoethyl)-*N*-methyloctanamide hydrochloride (NANMO) ^13^C NMR | 4 |
| **Figure S4** | *N*-(2-Aminoethyl)-*N*-methyloctanamide hydrochloride (NANMO) ^13^C NMR expansion | 6 |
| **Figure S5** | Kinetic mechanism fittings all models | 6 |
| **Figure S6** | Raw data for PA3944 enzymatic activity toward polymyxin B and NANMO | 7 |
| **Figure S7** | Ligand interaction maps for eight different conditions of NANMO docked into the PA3944 WT and E102A crystal structures | 8 |
| **Figure S8** | Eight poses of NANMO docked | 9 |
| **Scheme S1** | Hybrid ping-pong model derivation | 10-13 |
| **Table S1** | Percent frequency of interactions of docking poses | 14-15 |
| **Table S2** | Different types of GNAT chemical mechanisms and corresponding examples in the literature | 16 |


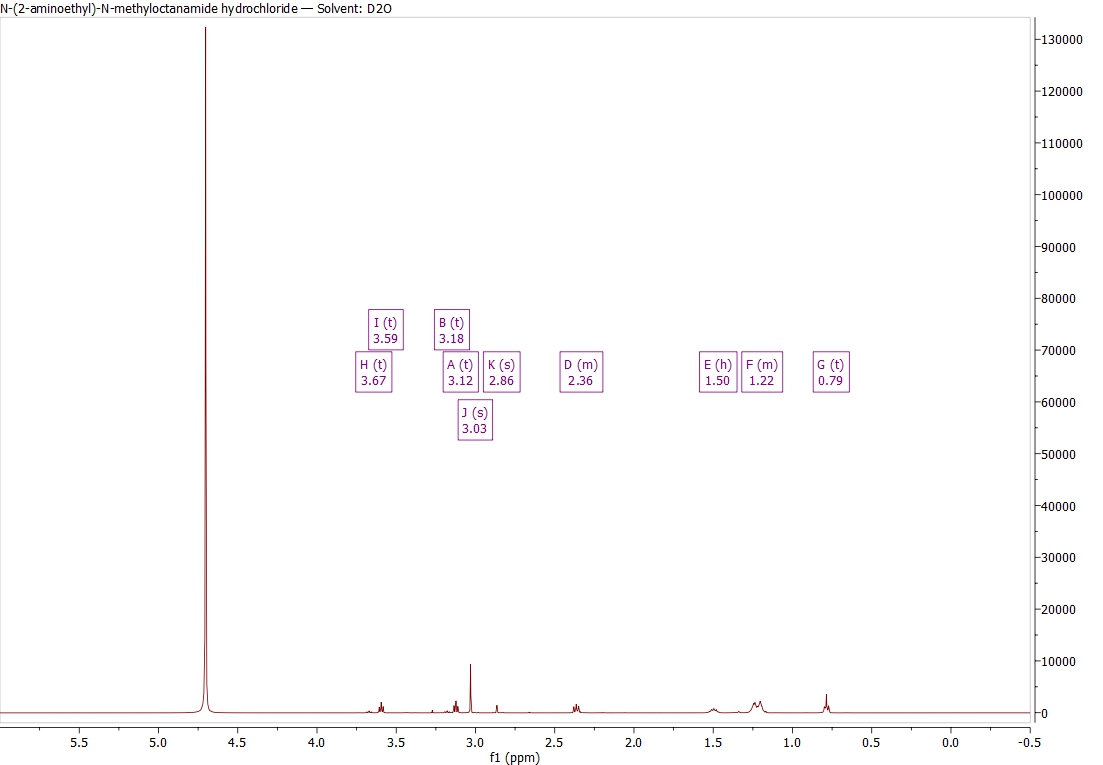


**Figure S1.** ^1^H NMR (D_2_O, 500 MHz) of *N*-(2-Aminoethyl)-*N*-methyloctanamide hydrochloride (NANMO).


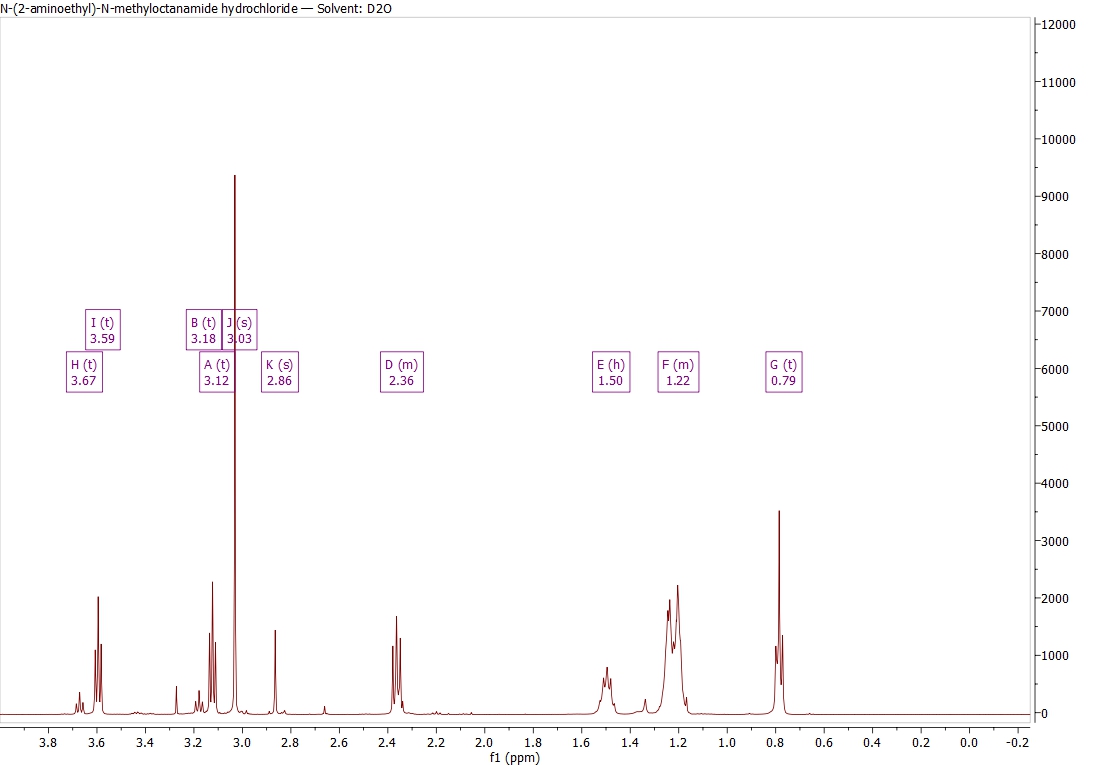


**Figure S2.** ^1^H NMR (D_2_O, 500 MHz) of *N*-(2-Aminoethyl)-*N*-methyloctanamide hydrochloride (NANMO) expansion.


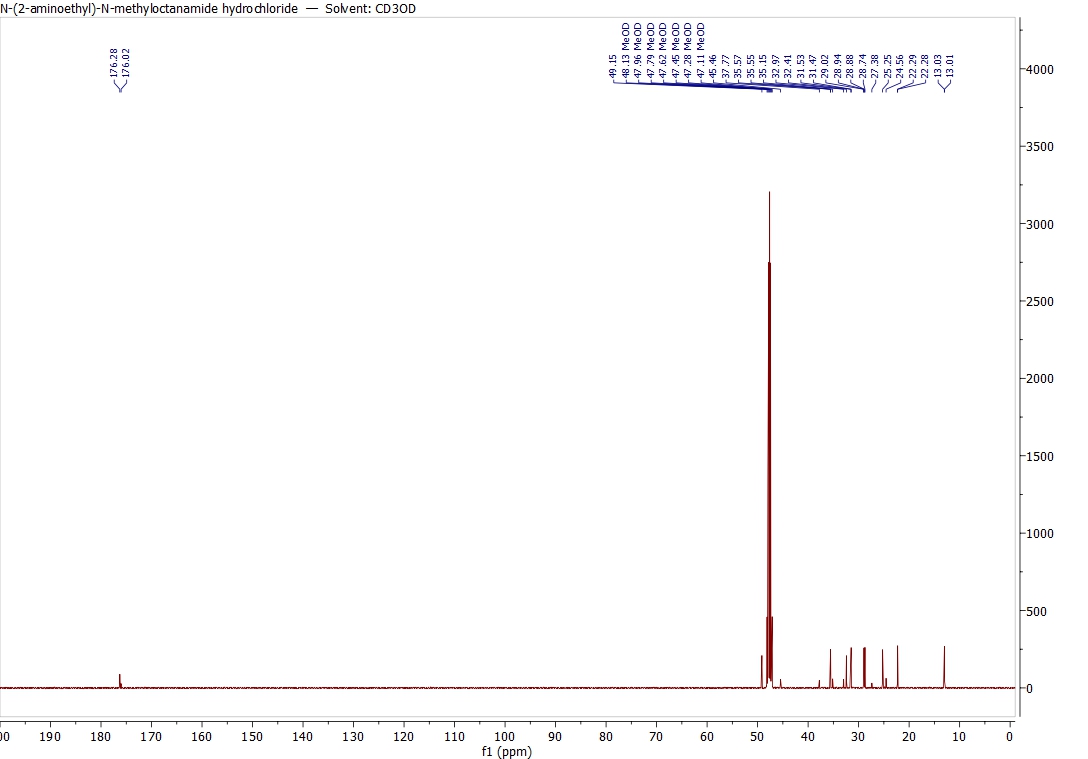


**Figure S3.** ^13^C NMR (D_2_O, 500 MHz) of *N*-(2-Aminoethyl)-*N*-methyloctanamide hydrochloride (NANMO).


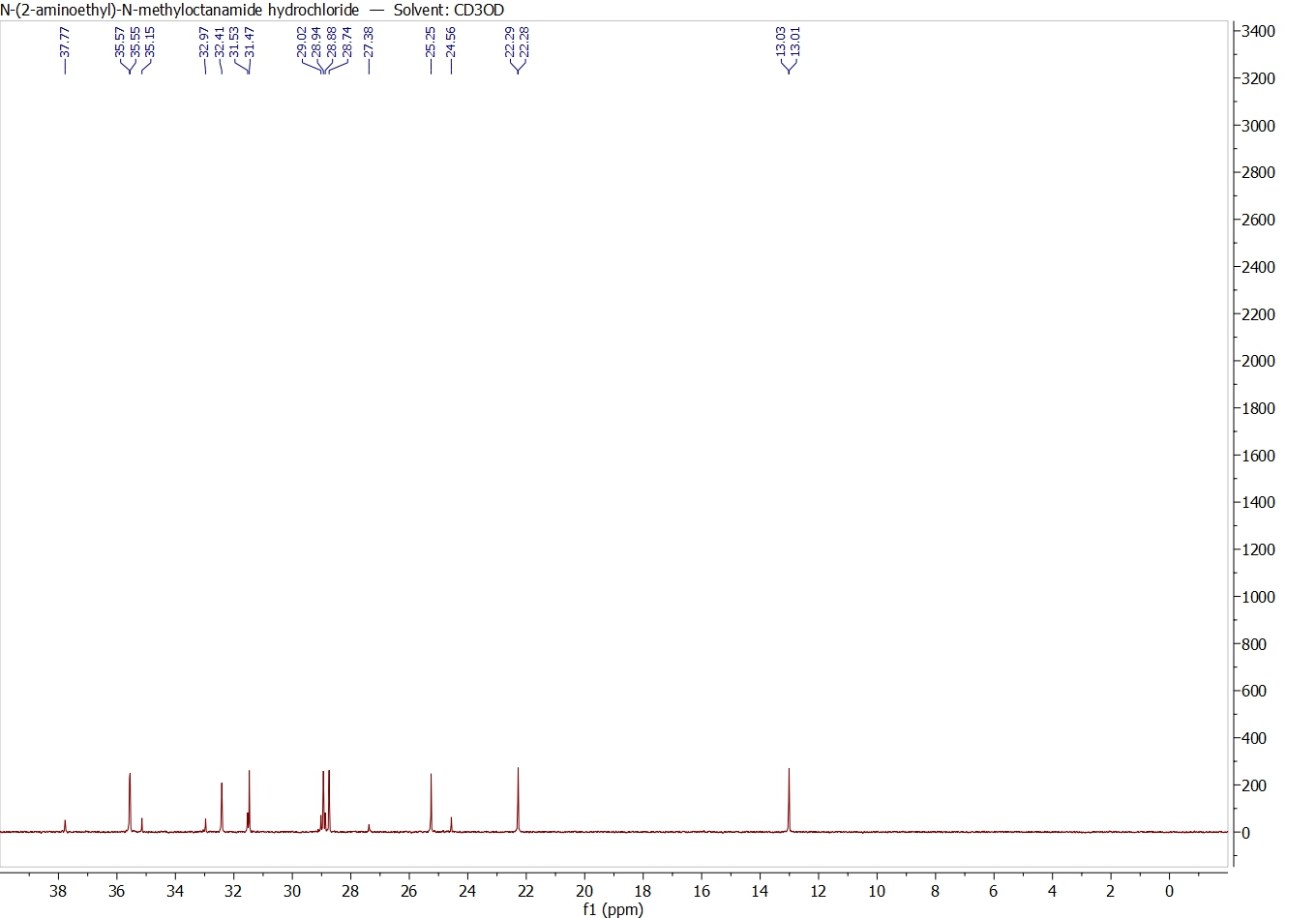


**Figure S4.** ^13^C NMR (CD_3_OD, 500 MHz) of *N*-(2-Aminoethyl)-*N*-methyloctanamide hydrochloride (NANMO) expansion

**
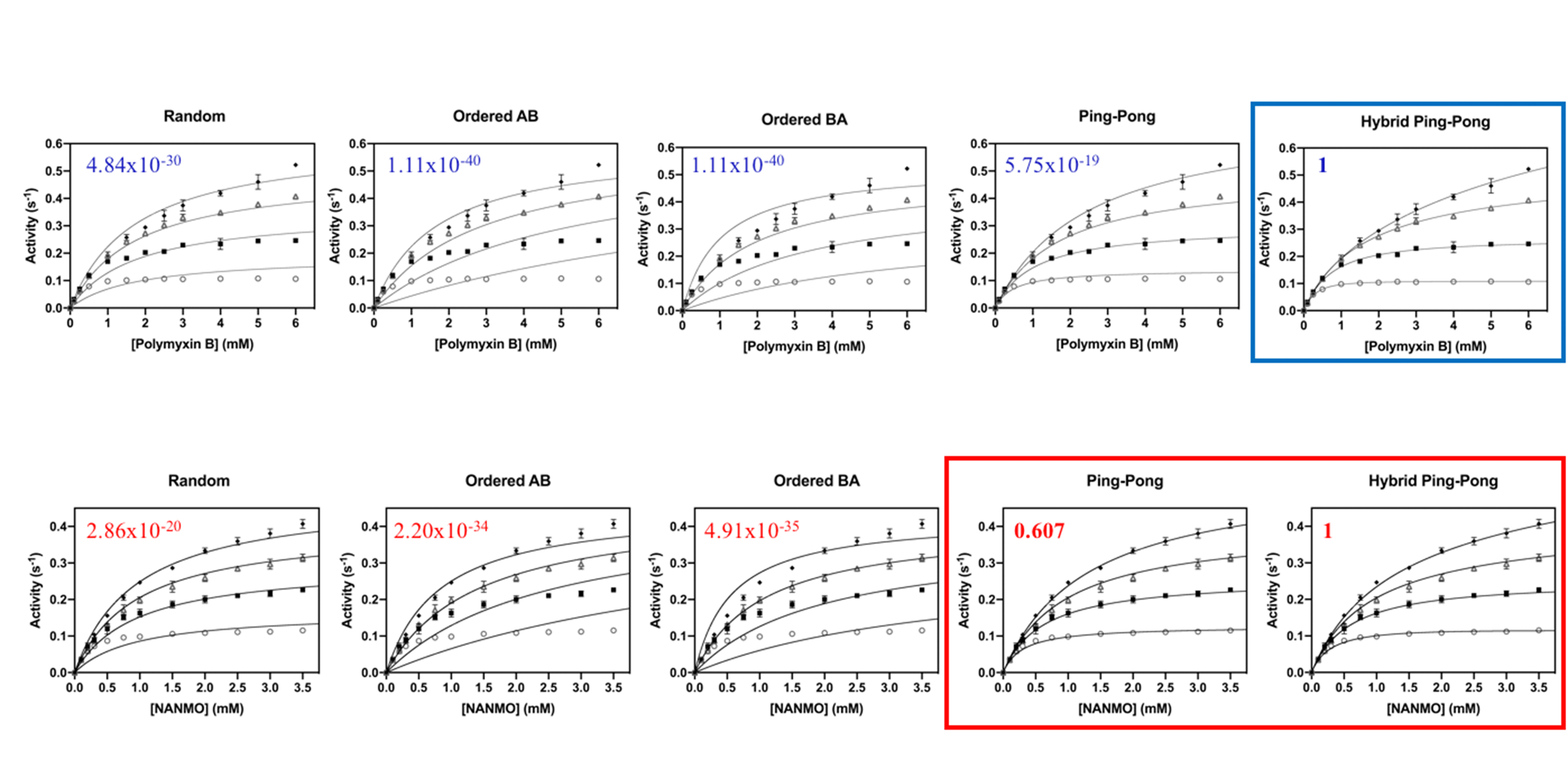
**

**Figure S5.** Kinetic mechanism fittings to five different models using two different acceptor substrates (Polymyxin B and NANMO). Relative likelihood values are shown inside plots and are color coded blue for Polymyxin B and red for NANMO. The plots containing the best model(s) fitting for each substrate are boxed.


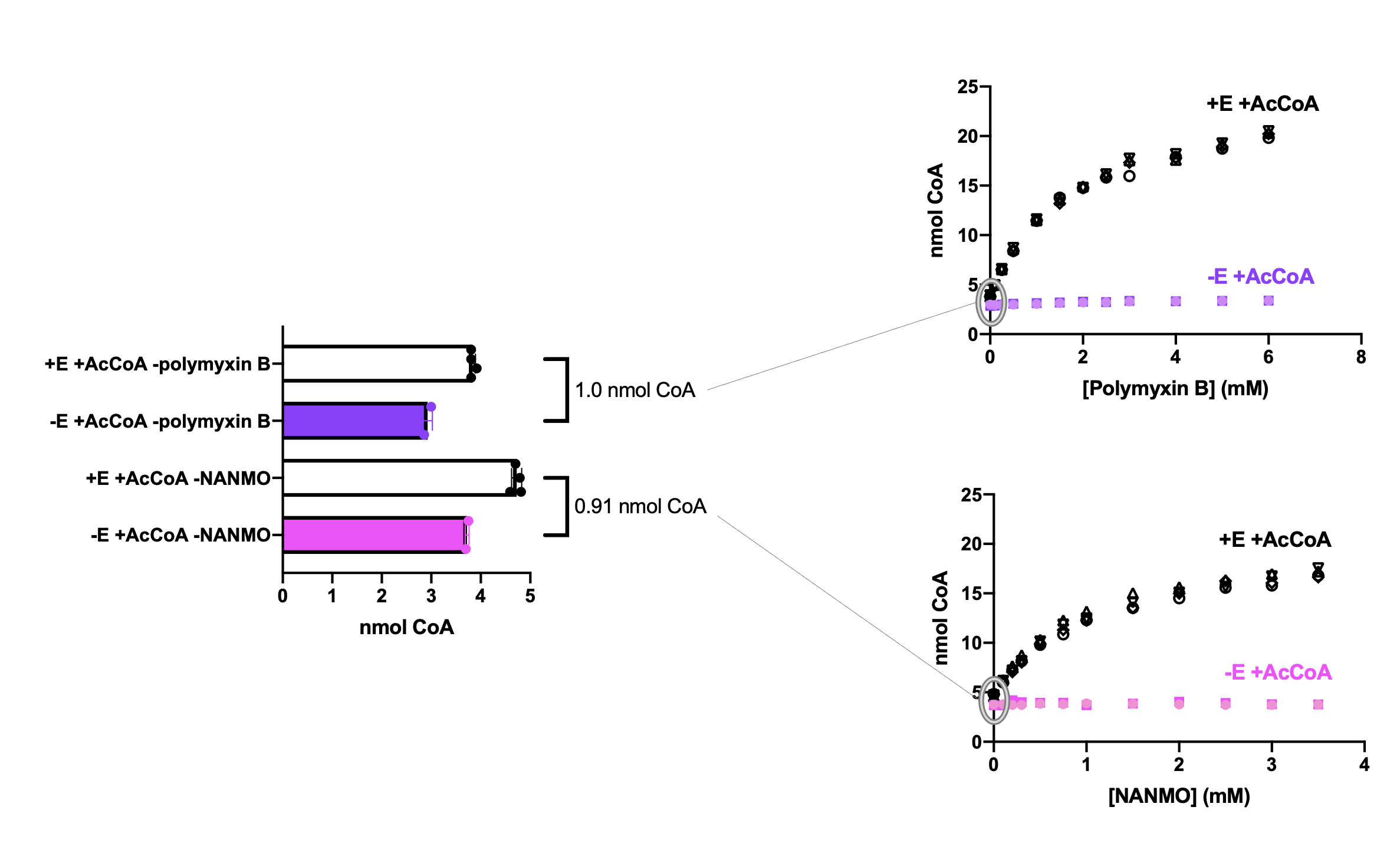


**Figure S6**. **Raw data for PA3944 enzymatic activity toward polymyxin B and NANMO.** Absorbances at 415 nm were converted to nmol CoA using the factor of 28 nmol/OD of CoA for each reaction. The scatter plots on the right show nmol of CoA produced as the concentration of polymyxin B or NANMO was increased from 0-6 or 0-3.5 mM, respectively. The concentration of AcCoA in each reaction was 0.5 mM AcCoA, and the amount of WT enzyme present in the reaction containing enzyme was 0.065 nmol. There is effectively no increase in CoA production as the concentration of acceptor substrates (polymyxin B or NANMO) is increased in absence of enzyme. Therefore, the observed increase of CoA in the presence of WT enzyme compared to reactions lacking enzyme can be attributed to acceptor substrate acetylation by the enzyme. The bar graph on the left shows the background nmol CoA present in reactions lacking acceptor substrates and in the presence and absence of WT enzyme. Thus, increased production of CoA compared to the control is attributed to AcCoA cleavage by the enzyme itself and may indicate acyl-enzyme intermediate formation; these are the data points shown circled in gray at x = 0 on the scatter plot. The difference in CoA production in the presence and absence of enzyme is shown adjacent to the bars (1.0 and 0.91 nmol CoA). Since the production of CoA is ~15 times greater than the amount of enzyme in the reaction, we cannot rule out the possibility that this additional CoA produced is due to other factors, including non-enzymatic acetylation of the protein, retention of CoA during purification that is released upon AcCoA introduction, or excess reducing agent or imidazole that was not completely removed during buffer exchange that reacts with DTNB. Further experiments are required to isolate the acyl-enzyme intermediate.


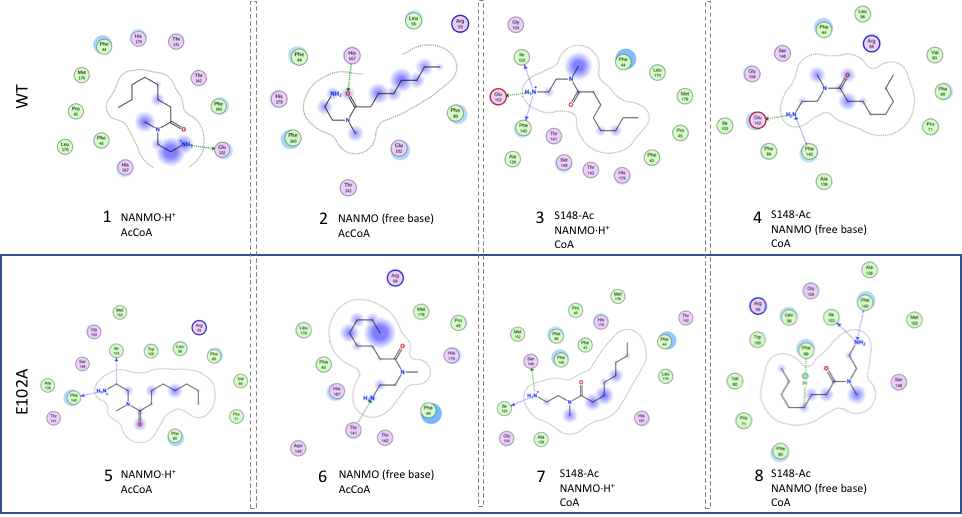


**Figure S7.** Ligand interaction maps for eight different conditions of NANMO docked into the PA3944 WT and E102A crystal structures. A single pose with the lowest binding energy for each of the eight experiments was selected. WT poses are numbered 1-4 and E102A are numbered 5-8. Ligands used for docking or enzyme modifications (e.g. acetylated S148) are noted by each diagram. Purple circles are polar residues, green circles are hydrophobic residues, purple circles with red border are acidic polar residues, purple circles with blue border are basic polar residues.


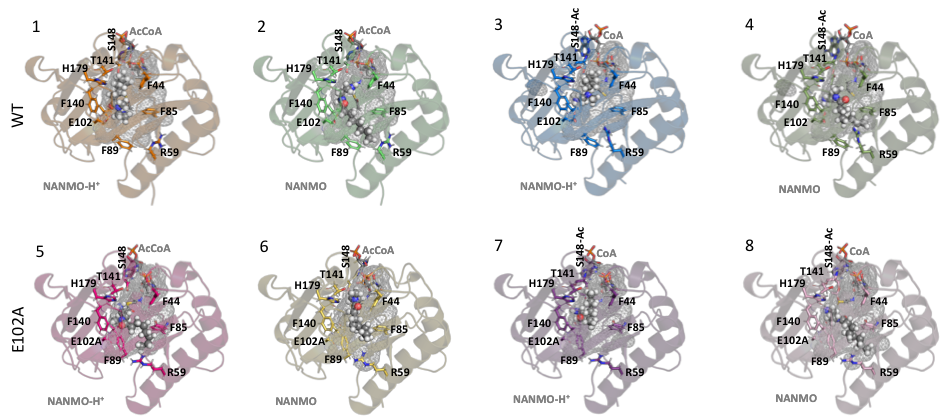


**Figure S8.** Eight poses of NANMO docked as described in **Figure S5**. Key residues are shown with sticks and the PA3944 protein is shown as ribbons. The acceptor site of the enzyme is shown with gray mesh and NANMO is in spheres with red as oxygen, blue as nitrogen, white as hydrogen and gray as carbon atoms. CoA is shown as gray sticks. NANMO does not occupy all of the acceptor site and adopts a variety of conformations.

**Scheme S1.** Derivation of a model of an enzyme-catalyzed reaction with two substrates and two products following a hybrid ping-pong/ordered mechanism

Given the following scheme

$$A+B \to P+Q$$

Following the reaction mechanism

The individual rate constants refer to the following half reactions

$$k_{1} A+E\to EA$$

$$k_{2} EA \to A + E$$

$$k_{3} EA\to P+EX$$

$$k_{4} B+EX \to EQ$$

$$k_{5} EQ\to B+EX$$

$$k_{6} EQ \to Q+E$$

$$k_{7} B+E \to EB$$

$$k_{8} EB\to B+E$$

$$k_{9} A+EB \to EAB$$

$$k_{10} EAB\to A+EB$$

$$k_{11} EAB\to P+EQ$$

Deriving the equation for the velocity of production of product P using the King-Altman method, we obtain:

$$v=\frac{n_{1}\left[ A \right]\left[ B \right]^{2}+n_{2} \left[ A \right]^{2}\left[ B \right]+n_{3}\left[ A \right]\left[ B \right]}{d_{1}\left[ A \right]\left[ B \right]^{2} + d_{2}\left[ A \right]^{2}\left[ B \right]+ d_{3}\left[ B \right]^{2}+ d_{4}\left[ A \right]\left[ B \right]+ d_{5}\left[ A \right]^{2} +d_{6}\left[ B \right]+d_{7} \left[ A \right]}$$

The individual factors in each of the terms are

$$n_{1}=k_{2}k_{4}k_{6}k_{7}k_{9}k_{11}+ k_{3}k_{4}k_{6}k_{7}k_{9}k_{11}$$

$$n_{2}= k_{1}k_{3}k_{4}k_{6}k_{9}k_{11}$$

$$n_{3}= k_{1}k_{3}k_{4}k_{6}k_{8}k_{10}+ k_{1}k_{3}k_{4}k_{6}k_{8}k_{11}$$

$$d_{1}= k_{2}k_{4}k_{7}k_{9}k_{11}+ k_{3}k_{4}k_{7}k_{9}k_{11}+ k_{2}k_{4}k_{6}k_{7}k_{9}+ k_{3}k_{4}k_{6}k_{7}k_{9}$$

$$d_{2}= k_{1}k_{4}k_{6}k_{9}k_{11}+ k_{1}k_{3}k_{4}k_{9}k_{11}$$

$$d_{3}= k_{2}k_{4}k_{6}k_{7}k_{11}+ k_{2}k_{4}k_{6}k_{7}k_{10}+ k_{3}k_{4}k_{6}k_{7}k_{11}+ k_{3}k_{4}k_{6}k_{7}k_{10}$$

$$d_{4}= k_{2}k_{4}k_{6}k_{9}k_{11}+ k_{3}k_{4}k_{6}k_{9}k_{11}+ k_{1}k_{4}k_{6}k_{8}k_{11}+ k_{1}k_{4}k_{6}k_{8}k_{10}+ k_{2}k_{5}k_{7}k_{9}k_{11}+ k_{3}k_{5}k_{7}k_{9}k_{11}+ k_{1}k_{3}k_{4}k_{8}k_{11}+ k_{1}k_{3}k_{4}k_{8}k_{10}$$

$$d_{5}= k_{1}k_{3}k_{6}k_{9}k_{11}+ k_{1}k_{3}k_{5}k_{9}k_{11}$$

$$d_{6}= k_{2}k_{4}k_{6}k_{8}k_{11}+ k_{2}k_{4}k_{6}k_{8}k_{10}+ k_{3}k_{4}k_{6}k_{8}k_{11}+ k_{3}k_{4}k_{6}k_{8}k_{10}$$

$$d_{7}= k_{1}k_{3}k_{6}k_{8}k_{11}+ k_{1}k_{3}k_{6}k_{8}k_{10}+ k_{1}k_{3}k_{5}k_{8}k_{11}+ k_{1}k_{3}k_{5}k_{8}k_{10}$$

Reordering and dividing each term by *n*_2_ we obtain the following equation

$$v=\frac{n_{1}^{'}\left[ A \right]\left[ B \right]^{2}+\left[ A \right]^{2}\left[ B \right]+n_{3}^{'}\left[ A \right]\left[ B \right]}{d_{1}^{'}\left[ A \right]\left[ B \right]^{2} + d_{2}^{'}\left[ A \right]^{2}\left[ B \right]+ d_{3}^{'}\left[ B \right]^{2}+ d_{4}^{'}\left[ A \right]\left[ B \right]+ d_{5}^{'}\left[ A \right]^{2} +d_{6}^{'}\left[ B \right]+d_{7}^{'} \left[ A \right]}$$

Where the factors in those terms are

$$n_{1}’ =\frac{n_{1}}{n_{2}}=\frac{\left( k_{2}+ k_{3} \right)k_{7}}{k_{1}k_{3}}$$

$$n_{2}’ = 1$$

$$n_{3}’=\frac{n_{3}}{n_{2}}=\frac{\left( k_{10}+ k_{11} \right)k_{8}}{k_{9}k_{11}}$$

$$d_{1}^{'}=\frac{d_{1}}{n_{2}}= \frac{\left( k_{11}+ k_{6} \right)\left( k_{2}+ k_{3} \right)k_{7}}{\left( k_{1}k_{3}k_{6}k_{11} \right)}$$

$$d_{2}^{'}=\frac{d2}{n2}= \frac{\left( k_{6}+ k_{3} \right)}{k_{3}k_{6}}$$

$$d_{3}^{'}=\frac{d_{3}}{n_{2}}= \frac{\left( k_{2}+ k_{3} \right)\left( k_{11}+ k_{10} \right)k_{7}}{k_{1}k_{3}k_{9}k_{11}}$$

$$d_{4}^{'}=\frac{d_{4}}{n_{2}}=\frac{\left( \left( k_{2}+ k_{3} \right)k_{9}+ k_{1}k_{8} \right)k_{4}k_{6}k_{11}+ \left( \left( k_{2}+ k_{3} \right)k_{5}k_{7}k_{9}+ k_{1}k_{3}k_{4}k_{8} \right)k_{11}+ \left( k_{6}+ k_{3} \right)k_{1}k_{4}k_{8}k_{10}}{k_{1}k_{3}k_{4}k_{6}k_{9}k_{11}}$$

$$d_{5}^{'}=\frac{d_{5}}{n_{2}}= \frac{\left( k_{6}+ k_{5} \right)}{\left( k_{4}k_{6} \right)}$$

$$d_{6}^{'}=\frac{d_{6}}{n_{2}}=\frac{\left( k_{2}+ k_{3} \right)\left( k_{11}+ k_{10} \right)k_{8}}{k_{1}k_{3}k_{9}k_{11}}$$

$$d_{7}^{'}=\frac{d_{7}}{d_{2}}=\frac{\left( k_{6}+ k_{5} \right)\left( k_{11}+ k_{10} \right)k_{8}}{k_{4}k_{6}k_{9}k_{11}}$$

With the purpose of simplifying and consolidating rate constants into parameters that could be fit, we defined the following parameters:

$$V_{m_{PP}}=\frac{k_{3}k_{6}}{k_{6}+ k_{3}}$$

$$V_{m_{Seq}}=\frac{k_{6}k_{11}}{k_{11}+ k_{6}}$$

$$\varepsilon_{A} = \frac{k_{1}k_{3}}{k_{2}+ k_{3}}$$

$$\varepsilon_{B}=\frac{k_{4}k_{6}}{k_{6}+k_{5}}$$

$$\varepsilon_{AB}= \frac{k_{9}k_{11}}{k_{10}+ k_{11}}$$

$$\gamma=\frac{k_{5}}{k_{6}+ k_{5}}$$

$$K_{d_{B}}=\frac{k_{8}}{k_{7}}$$

The parameter *V*_mPP_ represents the maximum velocity achievable through the Ping-Pong path, ignoring the product produced through the sequential path. Similarly, *V*_mSeq_ is the maximum velocity through the sequential path, ignoring what is produced by a ping-pong path. The parameter ε_A_ represents an analogous version of “catalytic efficiency” of an individual step, in this case involving the intermediate species EA. In a simple Michaelis-Menten scheme, that would be *k*_cat_/*K*_m_, which is the equivalent to$k_{1}k_{3}/(k_{2}+k_{3})$. The parameters ε_B_ and ε_AB_, are the similar parameters for the steps involving the enzyme species EB and EAB. The parameter γ is just a ratio to compare $k_{5}$ and$k_{6}$. *K*_dB_ is the dissociation constant of the binding of ligand B to the enzyme E.

Then:

$$n_{1}^{'}=\frac{k_{7}}{\varepsilon_{A}}$$

$$n_{2}^{'}= 1$$

$$n_{3}^{'}=\frac{k_{7}K_{d_{B}}}{\varepsilon_{AB}}$$

$$d_{1}^{'}=\frac{k_{7}}{\varepsilon_{A} V_{m_{Seq}}}$$

$$d_{2}^{'}=\frac{1}{V_{m_{PP}}}$$

$$d_{3}^{'}=\frac{k_{7}}{\varepsilon_{A}\varepsilon_{AB}}$$

$$d_{4}’ = \left( \frac{1}{\varepsilon_{A}} \right) \left( 1+\gamma\frac{k_{7}}{\varepsilon_{B}} \right)+ k_{7}\frac{K_{d_{B}}}{\varepsilon_{AB} V_{m_{PP}}}$$

$$d_{5}^{'}=\frac{1}{\varepsilon_{B}}$$

$$d_{6}^{'}= k_{7}\frac{K_{d_{B}}}{\varepsilon_{A}\varepsilon_{AB}}$$

$$d_{7}^{'}= k_{7}\frac{K_{d_{B}}}{\varepsilon_{B}\varepsilon_{AB}}$$

Replacing all the factors, the equation is:

$$v= \frac{\left( \frac{k_{7}}{\varepsilon_{A}} \right)\left[ A \right]\left[ B \right]^{2}+\left[ A \right]^{2}\left[ B \right]+\left( \frac{k_{7}K_{d_{B}}}{\varepsilon_{AB}} \right)\left[ A \right]\left[ B \right]}{\begin{aligned} \left( \frac{k_{7}}{\varepsilon_{A} V_{m_{Seq}}} \right)\left[ A \right]\left[ B \right]^{2} +\left( \frac{1}{V_{m_{PP}}} \right)\left[ A \right]^{2}\left[ B \right]+\left( \frac{k_{7}}{\varepsilon_{A}\varepsilon_{AB}} \right)\left[ B \right]^{2}+\left( \left( \frac{1}{\varepsilon_{A}} \right) \left( 1+ \gamma\frac{k_{7}}{\varepsilon_{B}} \right)+\frac{k_{7} K_{dB}}{\varepsilon_{AB} V_{m_{PP}}} \right)\left[ A \right]\left[ B \right] + \\ \left( \frac{1}{\varepsilon_{B}} \right)\left[ A \right]^{2} +\left( \frac{k_{7} K_{dB}}{\varepsilon_{A}\varepsilon_{AB}} \right)\left[ B \right]+\left( \frac{k_{7} K_{dB}}{\varepsilon_{B}\varepsilon_{AB}} \right)\left[ A \right] \end{aligned}}$$

If the enzyme species EQ tends to dissociate faster to produce Q rather than reverting to EX and releasing B, then $k_{5}\ll k_{6}$ and consequently$\gamma=0$. The equation simplifies to

$$v= \frac{\left( \frac{k_{7}}{\varepsilon_{A}} \right)\left[ A \right]\left[ B \right]^{2}+\left[ A \right]^{2}\left[ B \right]+\left( \frac{k_{7}K_{d_{B}}}{\varepsilon_{AB}} \right)\left[ A \right]\left[ B \right]}{\begin{aligned} \left( \frac{k_{7}}{\varepsilon_{A} V_{m_{Seq}}} \right)\left[ A \right]\left[ B \right]^{2} +\left( \frac{1}{V_{m_{PP}}} \right)\left[ A \right]^{2}\left[ B \right]+\left( \frac{k_{7}}{\varepsilon_{A}\varepsilon_{AB}} \right)\left[ B \right]^{2}+\left( \left( \frac{1}{\varepsilon_{A}} \right) +\frac{k_{7} K_{dB}}{\varepsilon_{AB} V_{m_{PP}}} \right)\left[ A \right]\left[ B \right] + \\ \left( \frac{1}{\varepsilon_{B}} \right)\left[ A \right]^{2} +\left( \frac{k_{7} K_{dB}}{\varepsilon_{A}\varepsilon_{AB}} \right)\left[ B \right]+\left( \frac{k_{7} K_{dB}}{\varepsilon_{B}\varepsilon_{AB}} \right)\left[ A \right] \end{aligned}}$$

**Table S1.** Percent frequency of interactions of NANMO with specific residues in PA3944 WT acceptor site. Values are based on 100 different docking poses; poses where the terminal amine of NANMO was not pointed toward the donor site or did not interact with residues in the acceptor site were excluded from the analysis. A total of 58, 90, 34, and 65 poses for Ser148-Ac-NANMO free base, Ser148-Ac-NANMO-H^+^, Ser148-NANMO free base, and Ser148-NANMO-H^+^ were used for this analysis. Ser148 is not modified, whereas Ser148-Ac is acetylated. Values are colored dark purple for >/ 80%, medium purple for >/ 60%, light purple for >/40%, gray for >/ 20% and white <20% conservation.

|  | S148(Ac) NANMO | S148(Ac) NANMO-H+ | AcCoA NANMO | AcCoA NANMO-H+ |  | Ave of all 4 |
| --- | --- | --- | --- | --- | --- | --- |
| Trp 23 | 0 | 3 | 6 | 0 |  | 2 |
| Phe 31 | 0 | 3 | 3 | 0 |  | 2 |
| Gln 39 | 0 | 0 | 9 | 0 |  | 2 |
| Val 40 | 0 | 1 | 9 | 0 |  | 2 |
| Met 41 | 0 | 1 | 9 | 6 |  | 4 |
| Glu 42 | 0 | 1 | 9 | 0 |  | 2 |
| Phe 43 | 86 | 86 | 71 | 78 |  | 80 |
| Phe 44 | 97 | 96 | 85 | 98 |  | 94 |
| Pro 45 | 36 | 42 | 53 | 71 |  | 51 |
| Ser 46 | 0 | 0 | 3 | 0 |  | 1 |
| Leu 48 | 0 | 0 | 12 | 12 |  | 6 |
| Gln 52 | 0 | 0 | 6 | 2 |  | 2 |
| Leu 56 | 24 | 19 | 29 | 26 |  | 25 |
| Val 57 | 0 | 1 | 0 | 0 |  | 0 |
| Arg 59 | 66 | 53 | 21 | 6 |  | 36 |
| Val 60 | 17 | 11 | 12 | 3 |  | 11 |
| Pro 71 | 53 | 42 | 18 | 8 |  | 30 |
| Phe 85 | 50 | 46 | 9 | 8 |  | 28 |
| Gly 87 | 24 | 13 | 0 | 0 |  | 9 |
| Leu 88 | 2 | 3 | 0 | 0 |  | 1 |
| Phe 89 | 59 | 43 | 62 | 35 |  | 50 |
| Asp 90 | 3 | 0 | 21 | 5 |  | 7 |
| Val 91 | 3 | 0 | 18 | 5 |  | 6 |
| Thr 92 | 2 | 0 | 15 | 2 |  | 4 |
| Met 93 | 3 | 2 | 47 | 23 |  | 19 |
| Glu 102 | 83 | 86 | 74 | 66 |  | 77 |
| Ile 103 | 24 | 29 | 9 | 8 |  | 17 |
| Gly 104 | 60 | 52 | 9 | 8 |  | 32 |
| Arg 106 | 16 | 22 | 3 | 20 |  | 15 |
| Trp 112 | 0 | 0 | 6 | 0 |  | 1 |
| Ala 139 | 7 | 7 | 9 | 5 |  | 7 |
| Phe 140 | 95 | 96 | 82 | 83 |  | 89 |
| Thr 141 | 81 | 78 | 44 | 71 |  | 68 |
| Thr 142 | 50 | 66 | 59 | 54 |  | 57 |
| Asn 145 | 60 | 53 | 38 | 25 |  | 44 |
| Ser 148 | 91 | 93 | 3 | 0 |  | 47 |
| His 167 | 59 | 57 | 79 | 92 |  | 72 |
| Leu 169 | 22 | 12 | 9 | 18 |  | 15 |
| Leu 170 | 34 | 37 | 21 | 46 |  | 34 |
| Met 176 | 12 | 19 | 21 | 31 |  | 21 |
| His 174 | 0 | 1 | 0 | 2 |  | 1 |
| His 179 | 95 | 84 | 85 | 95 |  | 90 |

**Table S2. Compilation of GNATs and their corresponding proposed catalytic mechanisms to date.** Note: some catalytic mechanisms assigned to each protein below are suggested and have not been shown conclusively. The abbreviations for proteins are also used in **Figure 9**. General acid/base denotes an enzyme that has residues in the active site that are directly participating as a general acid or base in the reaction. Water-mediated general acid/base means at least one water molecule is involved in mediating proton transfer to or from a single residue in the active site. Proton wire indicates the enzyme uses two or more water molecules, usually in a series, to mediate proton transfer to or from residues in the active site. PubMed IDs (PMIDs) are listed and correspond to the primary literature for each enzyme study.

| **Abbreviation** | **Full name** | **Catalytic Mechanism** | **Reference** |
| --- | --- | --- | --- |
| AAC(6')-Ii | Aminoglycoside 6'-*N*-acetyltransferase-Ii | Geometric Optimization | PMID: 14717599 |
| AAC-VIa | Aminoglycoside *N3-*acetyltransferase–VIa | Catalytic Triad | PMID: 29632894 |
| DAT | Dopamine *N-*acetyltransferase | Catalytic Triad | PMID: 22716280 |
| AAC(6')-Ib-cr | Aminoglycoside 6'-*N*-acetyltransferase-Ib and Ib-cr | General Acid/Base | PMID: 18710261 |
| GAT | Glyphosate *N-*acetyltransferase | General Acid/Base | PMID: 17272278 |
| Gcn5 | GCN5 histone acetyltransferase | Water-mediated general acid/base | PMID: 22574209 |
| MshD | Mycothiol synthase | Water-mediated general acid/base | PMID: 16326705 |
| AAC(2')-Ic | Aminoglycoside 2'-*N*-acetyltransferase-Ic | Water-mediated general acid/base | PMID: 12161746 |
| TTR | Tabtoxin resistance protein | Water-mediated general acid/base | PMID: 12527305 |
| SSAT | Spermidine/spermine acetyltransferase | Proton Wire | PMID: 18690703  PMID: 17516632 |
| SSPAT | Protein acetyltransferase | Proton Wire | PMID: 19473964 |
| PA4794 | PA4794 | Proton Wire | PMID: 24003232 |
| WecD | TDP-Fucosamine acetyltransferase | Proton Wire | PMID: 16855251 |
| iAANAT | Insect arylalkamine acetyltransferases | Proton Wire | PMID: 30094237 |
| SAT | Serotonin acetyltransferase | Proton Wire | PMID: 10319816 |
| PA3944 | PA3944 | Serine Nucleophile | This Paper |
| vPAT | Viral polyamine acetyltransferase | Serine Nucleophile (?)** | PMID: 22277659 |
| RimL | RimL | Serine Nucleophile (?) | PMID: 15817456 |
| MccE | Microcin C7 acetyltransferase | Serine Nucleophile (?) | PMID: 21507941 |
| BAT | Bleomycin acetyltransferase | Serine Nucleophile (?) | PMID: 19889644 |
| PaNAT | *Periplaneta americana* indolamine *N*-acetyltransferase | Serine Nucleophile (?) | * not in PubMed |
| CurA^AT^ | CurA GNAT-like Domain | ACP-Mediated | PMID: 31785925 |
| GphF^AT^ | GphF GNAT-like Domain | ACP-Mediated | PMID: 31785925 |
| SxtA^AT^ | SxtA GNAT-like Domain | ACP-Mediated | PMID: 29390180 |
| Ebony^CTD^ | Ebony GNAT-like Domain | ACP-Mediated | PMID: 30705105 |
| Mom | Adenine methylcarbamoyltransferase | Iron Radical | PMID: 32369169 |

*Sakamoto T, Ichihara N, Takeda M (1998) Characterization of indolamine *N*-acetyltransferase activity from the head ganglia of the American cockroach, *Periplaneta americana*. *Applied Entomology and Zoology* 33:97–104.

**Our analysis of the protein sequence (data not shown) indicates that a serine residue is conserved in a similar position as S148 in PA3944.
